# Inverse Isotope Effects in Single-Crystal to Single-Crystal Reactivity and the Isolation of a Rhodium Cyclooctane σ-Alkane Complex

**DOI:** 10.1021/acs.organomet.1c00639

**Published:** 2022-01-27

**Authors:** Laurence R. Doyle, Martin R. Galpin, Samantha K. Furfari, Bengt E. Tegner, Antonio J. Martínez-Martínez, Adrian C. Whitwood, Scott A. Hicks, Guy C. Lloyd-Jones, Stuart A. Macgregor, Andrew S. Weller

**Affiliations:** † Department of Chemistry, University of York, Heslington, York YO10 5DD, United Kingdom; ○ Department of Chemistry, University of Oxford, Physical and Theoretical Chemistry Laboratory, Oxford OX1 3QZ, United Kingdom; § Institute of Chemical Sciences, Heriot-Watt University, Edinburgh, Scotland EH14 4AS, United Kingdom; ∥ Department of Chemistry, Mansfield Road, University of Oxford, Oxford OX1 3TA, United Kingdom; ⊥ Department of Chemistry, University of Edinburgh, Edinburgh, Scotland EH9 3FJ, United Kingdom

## Abstract

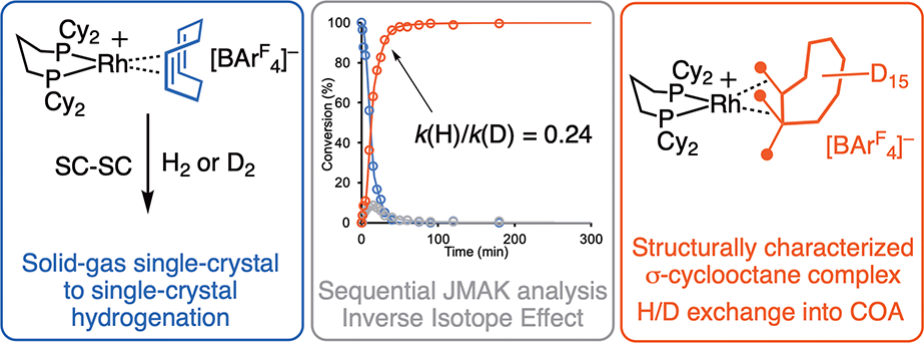

The sequential solid/gas single-crystal to single-crystal reaction of [Rh(Cy_2_P(CH_2_)_3_PCy_2_)(COD)][BAr^F^_4_] (COD = cyclooctadiene) with H_2_ or D_2_ was followed in situ by solid-state ^31^P{^1^H} NMR spectroscopy (SSNMR) and ex situ by solution quenching and GC-MS. This was quantified using a two-step Johnson–Mehl–Avrami–Kologoromov (JMAK) model that revealed an inverse isotope effect for the second addition of H_2_, that forms a σ-alkane complex [Rh(Cy_2_P(CH_2_)_3_PCy_2_)(COA)][BAr^F^_4_]. Using D_2_, a temporal window is determined in which a structural solution for this σ-alkane complex is possible, which reveals an η^2^,η^2^-binding mode to the Rh(I) center, as supported by periodic density functional theory (DFT) calculations. Extensive H/D exchange occurs during the addition of D_2_, as promoted by the solid-state microenvironment.

## Introduction

The isotopic substitution of hydrogen for deuterium is an invaluable tool for the study of the mechanism in synthesis and catalysis.^1−3^ Zero-point energy differences of E–H/D bonds (e.g., C–H/D) lead to changes in the temporal evolution of a reaction manifold if E–H bond activation, or formation, occurs at or before the rate-controlling step. This can be associated with a single transition state (a kinetic isotope effect, KIE) or preceding equilibria that result in a composite KIE (equilibrium isotope effect, EIE). While KIE or EIE normally act to slow the overall progress of a reaction when using the heavier isotopologue, an acceleration reflects an inverse isotope effect.^4^ While not always straightforward,^5,6^ this can be a result of EIE that favor productive intermediates in which D resides in a higher vibrational oscillator (i.e., C–D over M–D). The study of alkane C–H activation^3−5,7^ and the evidence for key, but fleeting in solution, σ-alkane intermediates^8,9^ have relied heavily on KIE or EIE effects.

**Scheme 1:**
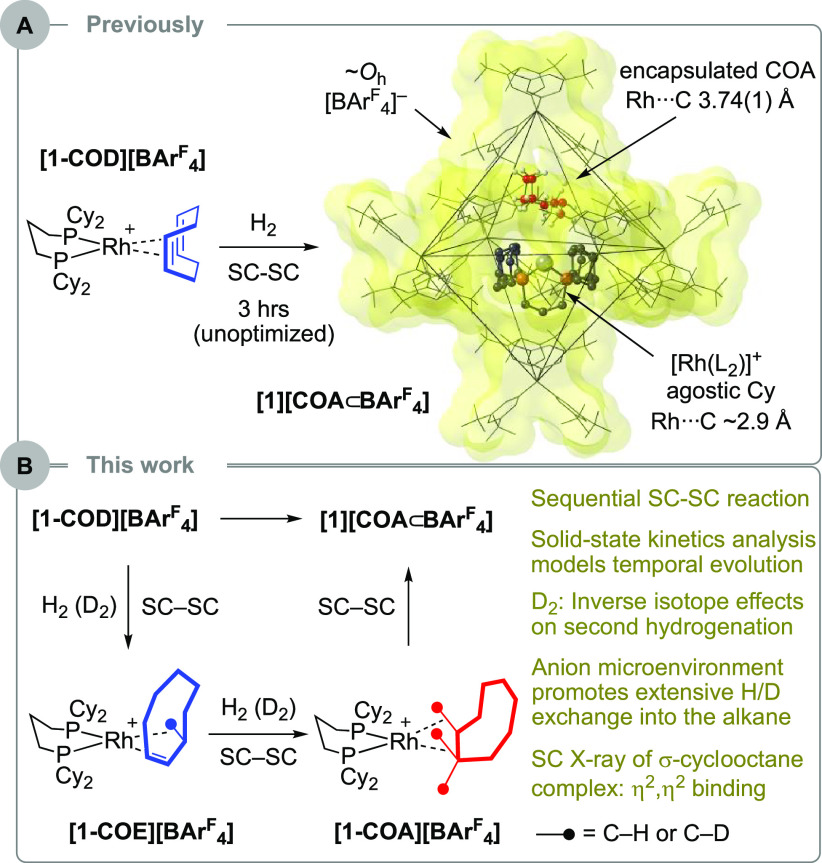
(A) Previously Reported SC–SC Hydrogenation of **[1-COD][BAr^F^_4_]** to Form **[1][COA⊂BAr^F^_4_]**; (B) This Work [**1**]^+^ = [Rh(L_2_)]^+^; L_2_ = Cy_2_PCH_2_CH_2_CH_2_PCy_2_.

We have previously reported on the use of in crystallo,^10^ solid-state molecular organometallic (SMOM) chemistry to isolate and characterize cationic σ-alkane complexes of Rh and Co by single-crystal to single-crystal (SC–SC) solid/gas hydrogenation of an alkene precursor.^11−13^ The secondary microenvironment provided by supporting [BAr^F^_4_]^−^ anions [Ar^F^ = 3,5-(CF_3_)_2_C_6_H_3_] is crucial in stabilizing weak 3-center 2-electron M···H–C bonds, meaning these complexes can be isolated and structurally characterized. However, for one precursor, [Rh(Cy_2_P(CH_2_)_3_PCy_2_)(COD)][BAr^F^_4_], **[1-COD][BAr**^**F**^_**4**_**]** (COD = cyclooctadiene), the formed alkane, cyclooctane (COA), does not remain bound to the metal when analyzed by X-ray crystallography after 3 h of hydrogenation.^14^ Instead, a Rh(I) cation with agostic^15^ interactions from the cyclohexyl groups is formed, with the liberated COA encapsulated in an octahedral array of [BAr^F^_4_]^−^ anions: **[1][COA⊂BAr**^**F**^_**4**_**]**, Scheme 1A. This multistep reaction involves sequential alkene hydrogenation and the loss of COA, presumably via an intermediate σ-cyclooctane complex. We now report that, by following the progress of this solid/gas reaction with H_2_ or D_2_, using a variety of methods, an inverse isotope effect is revealed, the leverage of which using D_2_ allows for the optimal temporal window to be determined for structural characterization of the intermediate σ-cyclooctane complex. Extensive H/D exchange at the alkane has also occurred, exchange that is promoted by the solid-state microenvironment. These observations add to the isotope effects previously reported in solid-state organometallic reactivity,^11,16−20^ which are still rare compared to those that occur in solution.^1,5,7^

## Results and Discussion

### Reaction with H_2_ and Reassignment of the Final Product in the Solid State

As discussed, we have previously shown that the addition of H_2_ to single crystals of **[1-COD][BAr**^**F**^_**4**_**]** for 3 h results in hydrogenation of the COD in a solid/gas reaction. The analysis of selected crystals, albeit weakly diffracting, by X-ray diffraction (Diamond Light Source, Beamline I19) provided a structural solution of **[1][COA⊂BAr**^**F**^_**4**_**]**.^14^ We now show that, while this host/guest motif is indeed formed, it is in fact an intermediate and extended reaction times with H_2_ result in an amorphous hydride-containing species as the final product. Nevertheless the overall reaction to form **[1][COA⊂BAr**^**F**^_**4**_**]** is a SC–SC^10^ transformation that hydrogenates COD to COA, presumably via a cyclooctene (COE) intermediate.

**Figure 1:**
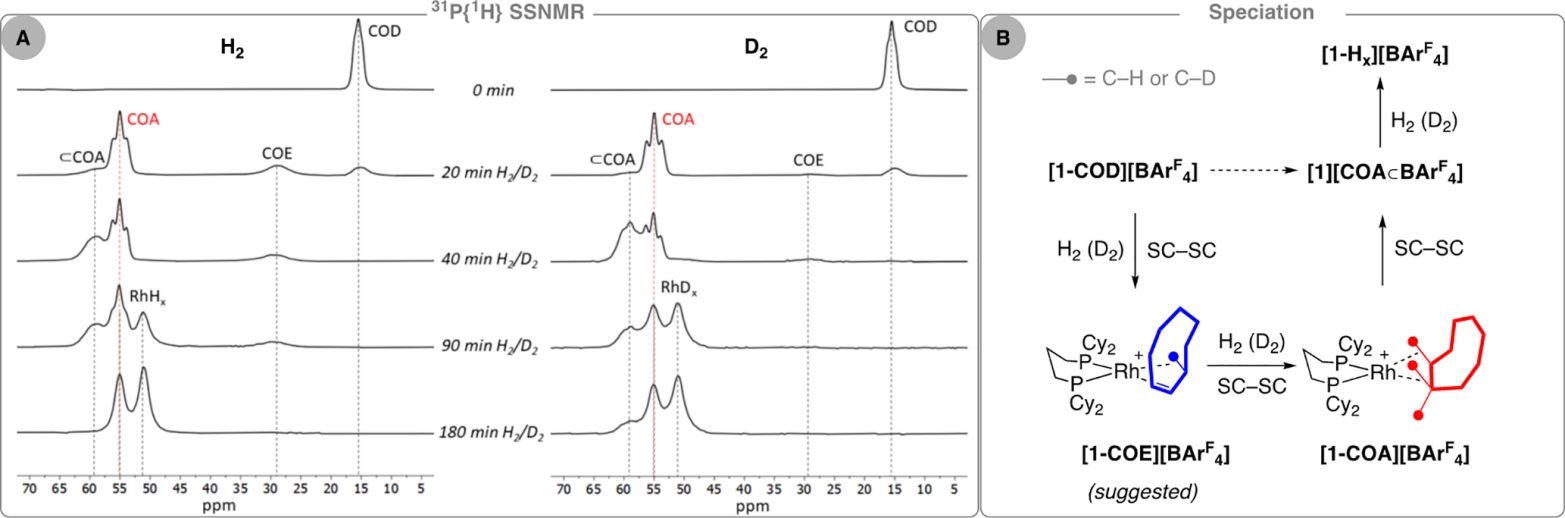
(A) Evolution of the products from the addition of H_2_ or D_2_ to crystalline **[1-COD][BAr**^**F**^_**4**_**]** (1.5 bar, 293 K), measured by ^31^P{^1^H} SSNMR spectroscopy (10 kHz spin rate, 273 K). Dotted lines to guide the eye. (B) Solid-state speciation.

The reaction was initially followed in situ on bulk samples of finely crushed and sieved **[1-COD][BAr**^**F**^_**4**_**]** (∼50 mg, 71–150 μm particle size) using solid-state ^31^P{^1^H} NMR spectroscopy (SSNMR). Repeated exposure of an uncapped solid-state rotor to H_2_ or D_2_ (1.5 bar, 293 K), capping, and analysis provided a temporal evolution for H_2_ and D_2_ addition, Figure 1A. This reveals speciation in which **[1-COD][BAr**^**F**^_**4**_**]** [δ 15.5, *J*(RhP) ∼ 120 Hz, apparent triplet due to crystallographically inequivalent P environments]^14^ is initially consumed to sequentially afford complexes assigned as **[1-COE][BAr**^**F**^_**4**_**]**, **[1-COA][BAr**^**F**^_**4**_**]**, **[1][COA⊂BAr**^**F**^_**4**_**]**, and finally hydride [Rh(Cy_2_P(CH_2_)_3_PCy_2_)H_*x*_][BAr^F^_4_], **[1-H**_***x***_**][BAr**^**F**^_**4**_**]**. The evolution of this system is first presented for H_2_ addition to baseline observations using D_2_ that are discussed later.

After 20 min of exposure of **[1-COD][BAr**^**F**^_**4**_**]** to H_2_, a new major species (∼70%) is observed at δ 55.0 [apparent triplet *J*(RhP) = 182 Hz]. The downfield shift and increased ^103^Rh–^31^P coupling constant (due to the weak trans influence σ-alkane ligand) identify this as a σ-alkane complex, [Rh(Cy_2_P(CH_2_)_3_PCy_2_)(COA)][BAr^F^_4_], **[1-COA][BAr**^**F**^_**4**_**]**, in comparison with other, well-defined, systems.^14,21^ Also observed, in similar proportions to one another (∼5–15%), are **[1-COD][BAr**^**F**^_**4**_**]** and broad signals at δ 28.9 and δ 58.9. On the basis of their chemical shifts and temporal evolution, these are assigned to [Rh(Cy_2_P(CH_2_)_3_PCy_2_)(COE)][BAr^F^_4_], **[1-COE][BAr**^**F**^_**4**_**]**, and **[1][COA⊂BAr**^**F**^_**4**_**]**, respectively. **[1-COE][BAr**^**F**^_**4**_**]** is proposed to have a structure as shown in Figure 1B (i.e., an alkene/agostic motif) on the basis of previously reported monoalkene complexes formed in solid/gas reactions, such as [Rh(Cy_2_P(CH_2_)_2_PCy_2_)(propene)][BAr^F^_4_].^22^ Such complexes can undergo rapid 1,3-hydrogen shifts (double bond isomerization) in the solid state, and it is likely that similar processes are operating for **[1-COE][BAr**^**F**^_**4**_**]**, vide infra. The encapsulated alkane complex **[1][COA⊂BAr**^**F**^_**4**_**]** has a bis-agostic structure in which two C–H···Rh interactions come from the cyclohexyl groups rather than an alkane^14^ and, thus, would be expected to show very similar chemical shifts and coupling constants to **[1-COA][BAr**^**F**^_**4**_**]** in the ^31^P{^1^H} SSNMR spectrum. While *J*(RhP) could not be resolved in this broad peak, the isotopologue formed using D_2_ does show an apparent, broad, triplet structure for the δ 59.9 signal [*J*(RhP) ∼ 190 Hz], similar to **[1-COA][BAr**^**F**^_**4**_**]** (Figure 1A, 40 min D_2_).

After 40 min of exposure to H_2_, the complete consumption of **[1-COD][BAr**^**F**^_**4**_**]** has occurred, **[1-COE][BAr**^**F**^_**4**_**]** is still observed but at a lower relative proportion, and **[1][COA⊂BAr**^**F**^_**4**_**]** has grown in. Initially surprising to us was that **[1][COA⊂BAr**^**F**^_**4**_**]** is not the final product. Further exposure to H_2_ resulted in the formation, after 3 h, of a new complex, identified by two signals at δ 51 and δ 55 with coupling to ^103^Rh not resolved, masked in the line width of the signals (fwhm = ∼335 Hz). In our initial report of the characterization of **[1][COA⊂BAr**^**F**^_**4**_**]** using SC–SC techniques, we correlated the structural solution (from weakly diffracting crystals selected from the reaction ensemble after 3 h of reaction) with these two signals in the ^31^P{^1^H} SSNMR spectrum.^14^ We now suggest this assignment was wrong and that these signals are instead due to a complex that has undergone further addition of H_2_ to form a Rh(III) complex of the general formula [Rh(Cy_2_P(CH_2_)_3_PCy_2_)H_*x*_][BAr^F^_4_], **[1-H**_***x***_**][BAr**^**F**^_**4**_**]**.

A number of observations support this new interpretation. (i) The dissolution of this final product in MeCN-*d*_3_ after the removal of excess H_2_ under vacuum forms the Rh(I) complex [Rh(Cy_2_P(CH_2_)_3_PCy_2_)(MeCN-*d*_3_)_2_][BAr^F^_4_]^23^ with the observation of dissolved H_2_ [δ 4.57] from reductive elimination^24^ and free COA (eq 1). (ii) The reduced magnitude of *J*(RhP) is indicative of a Rh(III) center. (iii) The formation of the hydride species in related systems by solid/gas reactivity has been reported previously.^19,25−27,29^ The rapid loss of H_2_ means we cannot comment on the precise number of hydrogen ligands, i.e., Rh(H)_2_ or Rh(H_2_)(H)_2_, or whether the Rh complex is still monomeric or has dimerized through bridging hydrides with the resulting loss of crystallinity.^25,26^ However, what is now clear is that this final species is *not***[1][COA⊂BAr**^**F**^_**4**_**]** as initially proposed. This highlights the potential problems associated with the analysis of a solitary single crystal by diffraction techniques and correlation with bulk analytical methods (e.g., NMR spectroscopy). As the final product, **[1-H**_***x***_**][BAr**^**F**^_**4**_**]** has lost long-range order (i.e., no discrete Bragg peaks); we suggest that, even though it is the only species observed by ^31^P{^1^H} SSNMR spectroscopy, manual crystal selection has a bias toward a very small proportion of **[1][COA⊂BAr**^**F**^_**4**_**]** that is still present; and thus our previously reported structural characterization.^14^
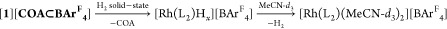
1

As the reaction with H_2_ up to the formation of **[1][COA⊂BAr**^**F**^_**4**_**]** retains crystallinity, guided by the ^31^P{^1^H} SSNMR data, we attempted to study the SC–SC reaction on suitably sized crystals (∼0.2 × ∼0.1 × ∼0.05 mm) at time points between 20 and 90 min using single crystal X-ray diffraction. While structural solutions could be found for the [BAr^F^_4_]^−^ anions, the metal fragments were heavily disordered, likely superpositions of **[1-COD][BAr**^**F**^_**4**_**]**, **[1-COE][BAr**^**F**^_**4**_**]**, **[1-COA][BAr**^**F**^_**4**_**]**, and **[1][COA⊂BAr**^**F**^_**4**_**]** in varying proportions.

### Reaction with D_2_ and the Crystallographic Characterization of a σ-Cycloalkane Complex

The solid/gas reaction of finely crushed and sieved **[1-COD][BAr**^**F**^_**4**_**]** with D_2_ was followed in situ using the same protocol as for H_2_. While this showed the equivalent set of sequential events occurring to ultimately form **[1-D**_***x***_**][BAr**^**F**^_**4**_**]**, Figure 1, a *qualitative* comparison of the evolution of the system provides insight into any isotope effects that are operating. First, **[1-COD][BAr**^**F**^_**4**_**]** is completely consumed in the same time scale as for H_2_ (40 min), suggesting that no (or small at best) isotope effect is operating for the first hydrogenation of COD. Second, **d**_***x***_**-[1-COE][BAr**^**F**^_**4**_**]** (where **d**_***x***_ denotes deuteration) is processed faster with D_2_, so at the 40 min time point, the solid mixture analyses show essentially only **d**_***x***_**-[1-COA][BAr**^**F**^_**4**_**]** and **d**_***x***_**-[1][COA⊂BAr**^**F**^_**4**_**]**. This suggests an inverse isotope effect is operating for the formation of **d**_***x***_**-[1-COA][BAr**^**F**^_**4**_**]** from **[1-COE][BAr**^**F**^_**4**_**]**. Finally, the system evolves to give the final product, **[1-D**_***x***_**][BAr**^**F**^_**4**_**]**, but its formation is slower with D_2_, as after 3 h, some **d**_***x***_**-[1][COA⊂BAr**^**F**^_**4**_**]** remains. This suggests a normal isotope effect is operating for the formation of this hydride species. These isotope effects will be discussed in more detail later.

**Figure 2:**
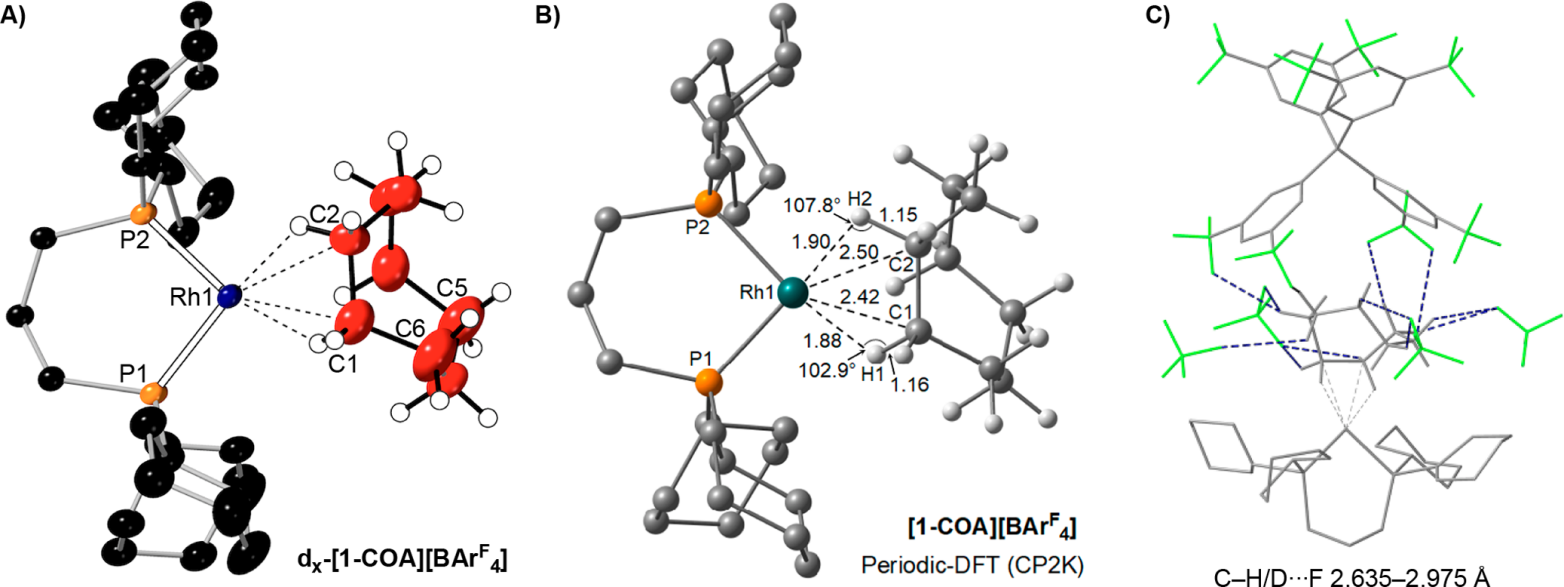
(A) Single-crystal X-ray structure of the cation in **d**_***x***_**-[1-COA][BAr**^**F**^_**4**_**]** (110 K, 30% displacement ellipsoids, selected H/D atoms shown in calculated positions). Selected bond lengths (Å) and angles (deg) **[1-COA][BAr**^**F**^_**4**_**]**: Rh–P1, 2.221(3); Rh–P2, 2.196(2); Rh–C1, 2.40(1); Rh–C2, 2.48(1); C1–C2, 1.49(2); C5–C6, 1.60(2) P1RhP2/RhC1C2 = 2.6(9). (B) Periodic DFT calculated structure of **[1-COA][BAr**^**F**^_**4**_**]**. (C) Orientation of the cation and CF_3_ groups from proximal anions highlighting the C–H/D···F noncovalent interactions.

This reaction was repeated with D_2_ on larger single crystalline material. By optimization of the time of D_2_ addition a structural solution for **d**_***x***_**-[1-COA][BAr**^**F**^_**4**_**]** could be obtained after 40 min using single-crystal X-ray diffraction.^28^ While the data analysis was complicated by pseudomerohedral twinning and superpositionality disordered minor components of **[1-COD][BAr**^**F**^_**4**_**]** and **d**_***x***_**-[1-COE][BAr**^**F**^_**4**_**]**, the structural solution was unambiguous (*R* = 7.15%) and revealed a [Rh(Cy_2_PCH_2_CH_2_CH_2_PCy_2_)]^+^ fragment bound with a cyclooctane ligand, Figure 2A. This successful structural solution relied on the combination of isotope effects operating, as discussed above, that favor **d**_***x***_**-[1-COA][BAr**^**F**^_**4**_**]** being formed in a compositionally purer form compared to that with H_2_ addition. Full details of the refinement can be found in the Supporting Information.

The cation in **d**_***x***_**-[1-COA][BAr**^**F**^_**4**_**]** has a pseudo square planar Rh(I) center, which is coordinated on one side by the chelating phosphine [Rh–P = 2.221(3), 2.196(2) Å] and the other by the cyclooctane ligand. The C–C bond distances in the COA ligand were unrestrained and fall in the range of 1.44(2)–1.60(2) Å, consistent with single bonds and confirming full hydrogenation of the COD. The COA ligand engages in a 1,2-motif σ-bond interaction with rhodium, as signaled by the Rh···C distances from adjacent C atoms [C1, C2; 2.40(1), 2.48(1) Å], being similar to those found for other alkane ligands that bind in a 1,2-motif with this, or related, Rh-fragments, e.g., norbornane [2.408(2), 2.402(2) Å],^14^ isobutane [2.36(2), 2.442(7) Å],^11^ and 2-methylbutane [2.348(9), 2.39(1) Å].^13^ Hydrogen (deuterium) atoms were not located. Periodic density functional theory (DFT) calculations (Figure 2B) reproduce the structure well, capturing the slight asymmetry in the Rh···C distances [calc. 2.43 and 2.50 Å] and highlight the elongation of the C–H bonds engaged in the C–H → Rh σ-interactions (ca. 1.15 Å). They also show that the COA ligand binds in an η^2^,η^2^ motif, [Rh(Cy_2_PCH_2_CH_2_CH_2_PCy_2_)(η^2^,η^2^-COA)][BAr^F^_4_] as found for the norbornane analogue.^14^ The Rh···C distances are shorter than that found in the Rh(III) complex [RhH(κ^3^-Cy_2_P(CH_2_)_2_CH(CH_2_)_2_PCy_2_)(COA)][BAr^F^_4_], 2.90(3) Å, where a η^1^-coordination mode is observed for COA. The alkane ligand in **d**_***x***_**-[1-COA][BAr**^**F**^_**4**_**]** sits in a pocket defined by the proximal [BAr^F^_4_]^−^ anions, Figure 2C, and there are a number of relatively close C–H···F contacts that act to further stabilize the complex, as described before for other alkane complexes of this type.^13,29,30^ QTAIM, noncovalent interaction plots, and NBO analyses support the assigned hapticity and microenvironment effects (Supporting Information).

Cyclooctane complexes have been identified as early intermediates in C–H activation, using fast time-resolved infrared techniques, having lifetimes on the ns−μs time scale, e.g., Rh(η^5^-C_5_Me_5_)(CO)(cyclooctane)^31^ and Tp*Rh(CNR)-(cyclooctane).^32^ σ-Alkane complexes of cyclooctane have also been identified as intermediates in alkane dehydrogenation reactions using computational methods.^33^ The isolation of **[1-COA][BAr**^**F**^_**4**_**]** thus represents a structurally authenticated example that has a significant lifetime at 293 K. Attempts to characterize **[1-COA][BAr**^**F**^_**4**_**]** or **[1-COE][BAr**^**F**^_**4**_**]** by low temperature solution NMR spectroscopy^8,34,35^ (CD_2_Cl_2_, 183 K) led to the formation of intractable solids.

Exposure of single crystals of **[1-COD][BAr**^**F**^_**4**_**]** to D_2_ for a total of 60 min and analysis of selected crystals by single crystal X-ray diffraction resulted in a structural refinement that confirmed the formation of **d**_***x***_**-[1][COA⊂BAr**^**F**^_**4**_**]** (Supporting Information), but due to a drop off in data quality, alongside significant superpositional disorder, this only provided atom connectivity. Nevertheless, this confirms the previous report of the formation of this complex in a SC–SC reaction.^14^

### Quantification of the Isotope Effects in the Solid/Gas Reaction Using Johnson–Mehl–Avrami–Kologoromov (JMAK) Analysis

**Figure 3:**
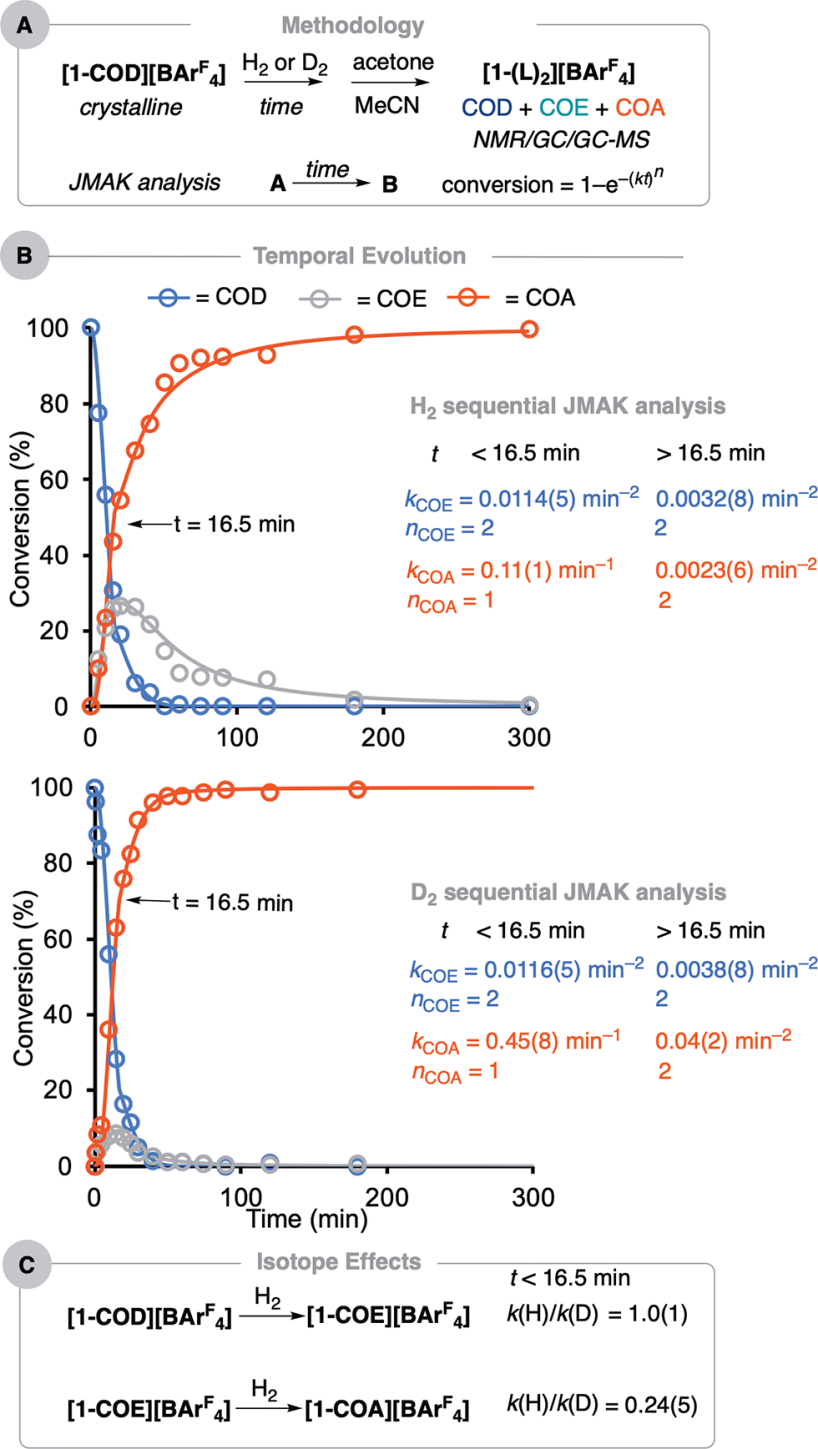
(A) Methodology for temporal analysis (L = MeCN-*d*_3_ or acetone-*d*_6_) and simple JMAK analysis. (B) Temporal evolution: each data point to a separate experiment (1.5 bar H_2_ or D_2_, 293 K); sequential JMAK analysis fits with growth rate (*k*) and Avrami (*n*) constants: *k*/*n*_COE_ refers to [COD]_total_ to [COE]_total_; *k*/*n*_COA_ refers to [COE]_total_ to [COA]_total_; they do not reflect actual speciation. (C) Calculated isotope effects for the SC–SC organometallic transformation before *t* = 16.5 min.

The time course of these solid/gas reactions was followed using solution quenching experiments that determine the relative ratios of COD, COE, and COA. Starting from **[1-COD][BAr**^**F**^_**4**_**]**, the same method described for the ^31^P{^1^H} SSNMR experiments was used for individual samples that were exposed, over incrementally longer reactions times, to either H_2_ or D_2_ in NMR tubes (Figure 3A, 7.6 mg each sample, 1.5 bar, 293 K). Each of these was quenched by evacuation of the tube, refilling with Ar, and then addition of a suitable coordinating solvent. Using acetone-*d*_6_, a mixture of [Rh(Cy_2_P(CH_2_)_3_PCy_2_)(acetone-*d*_6_)_2_][BAr^F^_4_],^23^ displaced COE and COA, and unreacted **[1-COD][BAr**^**F**^_**4**_**]** were formed. Analysis by solution ^31^P{^1^H} and ^1^H NMR spectroscopy allowed the ratios of COD, COE, and COA to be determined by integration relative to [BAr^F^_4_]^−^. Further addition of MeCN-*d*_3_ to these solutions liberated bound COD, forming [Rh(Cy_2_P(CH_2_)_3_PCy_2_)(MeCN-*d*_3_)_2_][BAr^F^_4_],^23^ and the resulting COA/COE/COD ensemble was analyzed using GC-MS for H_2_ and D_2_ additions. Both methods give very similar temporal profiles for H_2_ addition, but GC-MS-derived data allow for quantification of both H_2_ and D_2_ addition without interference from additional H/D exchange processes (vide infra) that affect analysis by ^1^H NMR spectroscopy.^36^ These data were then used as a proxy for the organometallic solid-state reactivity that is occurring. As this methodology determines [COA]_TOTAL_, and does not discriminate between bound and free alkane, it reports on the ensemble of **[1-COA][BAr**^**F**^_**4**_**]**, **[1][COA⊂BAr**^**F**^_**4**_**]**, and **[1-H**_***x***_**][BAr**^**F**^_**4**_**]**. However, the rate of change of [COA]_TOTAL_ formation describes **[1-COA][BAr**^**F**^_**4**_**]**, as this is the first formed species in this set.

Figure 3B presents the resulting reaction course plots for H_2_ and D_2_ addition over a 5 h sampling period. Qualitatively, both show the same rate of consumption of **[1-COD][BAr**^**F**^_**4**_**]** that is complete after 40 min. COE is observed to be formed as an intermediate, but its relative maximum is lower; COA is formed faster for D_2_ addition. This signals faster progress of COE to COA using D_2_, i.e., an inverse isotope effect as suggested from the complementary ^31^P{^1^H} SSNMR experiments described earlier.

The same batch of sieved crystalline material was used for each of the individual H_2_ and D_2_ experiments shown in Figure 3B. The repetition of selected data points using a different batch of crystalline materials (Supporting Information) showed a small amount of variability between batches, but the data are still fully consistent with the overall temporal profiles recorded for the main experiments. This may be due to surface area effects for different crystalline batches or other experimental variables (e.g., small changes in the pressure of H_2_ or D_2_).

These data have been analyzed using a sequential Johnson–Mehl–Avrami–Kologoromov (JMAK)^37,38^ solid-state kinetic model for an A → B → C reaction sequence (see the Supporting Information for full derivation and implementation). Figure 3B shows the resulting fits (solid lines). JMAK analysis describes the progress of a solid-state reaction, i.e., A → B, by a nucleation and growth model, where *k* is the growth rate constant and *n* is the Avrami exponent, eq 2. Exponents close to *n* = 2, 3, and 4 have been suggested to be due to 1-D, 2-D, and 3-D reaction growth dimensionality, respectively, while *n* = 1 suggests a noncooperative process and can be related to classical first order processes in homogeneous systems.^39^ JMAK analysis has been used to model simple SC–SC, A → B, processes.^11,13,40−42^
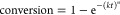
2At the early stages of the reaction (<16.5 min), the values of *k* and *n* are the same within error for both H_2_ and D_2_ addition to **[1-COD][BAr**^**F**^_**4**_**]** (*k* = 0.0114(5) and 0.0116(5) min^–2^ respectively; *n* = 2). Thus, there is no isotope effect observed for the hydrogenation of **[1-COD][BAr**^**F**^_**4**_**]** to give **[1-COE][BAr**^**F**^_**4**_**]**, i.e., *k*(H)/*k*(D) = 1, Figure 3C. As the second addition of D_2_ to **[1-COE][BAr**^**F**^_**4**_**]** is faster than that with H_2_ (vide infra, Supporting Information) and the formation of **[1-H**_***x***_**][BAr**^**F**^_**4**_**]** qualitatively shows a normal isotope effect, we suggest that hydrogenation is not diffusion limited. Rate-limiting substrate diffusion has been demonstrated for in crystallo organometallic reactivity in MOFs,^17^ while inverse isotope effects have been noted for diffusion of H_2_ or D_2_ into microporous materials.^43^ The structurally very close complex [Rh(Cy_2_P(CH_2_)_3_PCy_2_)(NBD)][BAr^F^_4_] (NBD = norbornadiene) undergoes rapid SC–SC hydrogenation to form the corresponding σ-alkane complex in <5 min (cf. **[1-COD][BAr**^**F**^_**4**_**]** 40 min),^14^ which also argues against rate limiting diffusion of H_2_. Instead, we propose a rate-limiting, possibly correlated, intramolecular dissociation of one of the alkene groups in COD. Pertinently, in solution, [Rh(chelating phosphine)(COD)]^+^ complexes also undergo hydrogenation a lot slower than their NBD analogues although the reasons behind this are not clear.^44^

This model is complicated by a subtle point of discontinuity at *t* = 16.5 min for both H_2_ and D_2_ addition, which when included provides a better fit to the data. This results in a reduced value of *k* for *both* H_2_ (0.0032(8) min^–2^) and D_2_ (0.0038(8) min^–2^) addition to **[1-COD][BAr**^**F**^_**4**_**]** after 16.5 min that are the same within error, with no change in *n*, and thus no measurable isotope effect. As this change occurs at the same point in time for *both* H_2_ and D_2_ addition, we suggest this is not an experimental artifact and is triggered at a certain conversion of **[1-COD][BAr**^**F**^_**4**_**]**. As the microcracking of single crystals^45^ would be expected to *increase* the rate of conversion through surface area arguments, we speculate that this change has to do with a correlated,^46^ but subtle, change in the spatially averaged periodic structure that occurs in the hydrogenation of **[1-COD][BAr**^**F**^_**4**_**]**. The repetition of these experiments on larger single crystals and the measurement of the unit cell parameters with time showed no significant step change in axes lengths that would signal a phase change.

In contrast to the consumption of **[1-COD][BAr**^**F**^_**4**_**]**, the subsequent hydrogenation of **[1-COE][BAr**^**F**^_**4**_**]** is significantly faster for D_2_ addition than that of H_2_ (*k* = 0.45(8) vs 0.11(1) min^–1^, respectively) at the initial stages of the reaction, and the Avrami exponent is now unity for both. There is thus an inverse isotope effect observed: *k*(H)/*k*(D) = 0.24(5). After 16.5 min, *k* again decreases significantly, but *n* is now 2, there is also a significant inverse isotope effect, *k*(H)/*k*(D) = 0.06(3) (*k* = 0.0023(6) vs 0.04(2) min^–2^). The change in “dimensionality”, *n*, makes a direct comparison difficult between the two regimes. Interestingly, in line with Finke’s suggestion that *k* and *n* are convoluted and cannot be easily separated,^38^ for this *n* = 2 regime, [*k*(H)]^1/2^/[*k*(D)]^1/2^ = 0.24(5), which is the same as for the pre-16.5 min value (*n* = 1). The consequence of these combined inverse isotope effects for **[1-COE][BAr**^**F**^_**4**_**]** hydrogenation is that after 40 min the conversion of COD to COA is essentially complete using D_2_, but considerable (∼20%) COE still remains when using H_2_.

### H/D Exchange in COE and COA and the Inverse Isotope Effect

**Figure 4:**
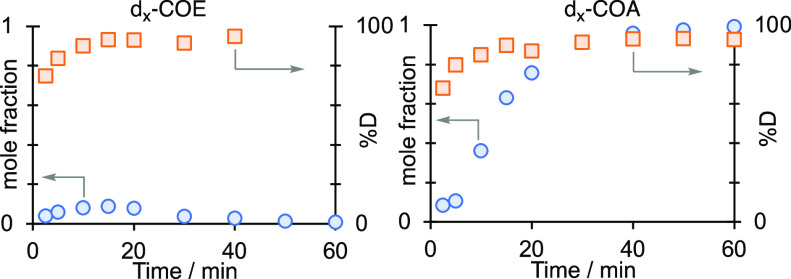
% H/D incorporation into COE or COA from the addition of D_2_ (1.5 bar) to crystalline **[1-COD][BAr**^**F**^_**4**_**]**, measured by GC-MS. ○ = mole fraction of COE or COA_TOTAL_. □ = %D incorporation relative to the maximum possible. COE D incorporation data not available after 50 min due to weak signal-to-noise at very low COE mole fractions.

The evolution of the reaction between **[1-COD][BAr**^**F**^_**4**_**]** and D_2_ was monitored using GC-MS on the same finely powdered samples as that used for the quenching experiments. This showed that significant, almost complete, H/D exchange was occurring into both COE and COA in this SC–SC solid/gas reaction. No H/D exchange was observed into COD. Figure 4 shows the resulting time course versus %D incorporation for COE and COA_TOTAL_ using D_2_. After 40 min, the remaining COE reaches ∼95% D incorporation with a weighted average of ∼d_13_-COE. High levels of exchange (∼75% D, ∼d_11_-COE) are achieved at the first measured time point of 2.5 min. H/D exchange into **[1-COE][BAr**^**F**^_**4**_**]** thus occurs rapidly. The temporal profile for H/D exchange into COA follows closely with that of COE and not [COA]_TOTAL_, resulting in 68% D (∼d_11_-COA) after 2.5 min and rising to 93% D incorporation after 40 min. This suggests a formulation of ∼**d**_**15**_**-[1-COA][BAr**^**F**^_**4**_**]** for the crystallographically characterized sample (Figure 2A).

**Scheme 2:**
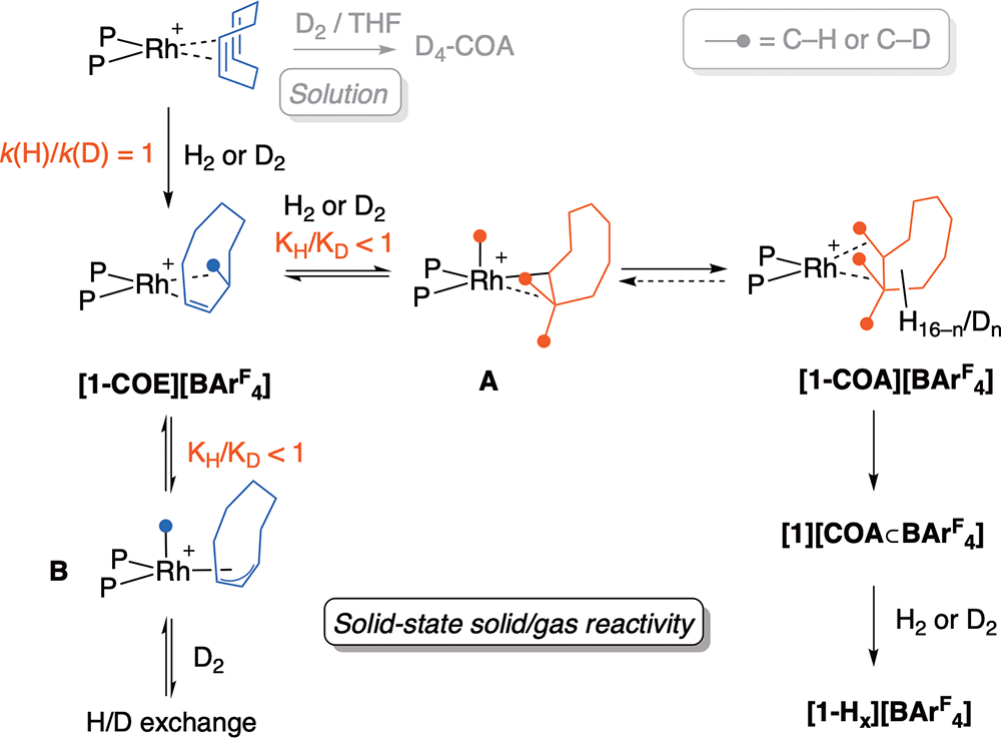
Suggested Mechanism for H/D Exchange in the Solid State [BAr^F^_4_] anions are not shown.

A plausible mechanism for this SC–SC H/D exchange process is shown in Scheme 2; this may also explain the inverse isotope effect observed for the formation of **[1-COA][BAr**^**F**^_**4**_**]**.

The initial, irreversible, addition of H_2_/D_2_ to **[1-COD][BAr**^**F**^_**4**_**]** results in **[1-COE][BAr**^**F**^_**4**_**]** for which no isotope effect is measured. The subsequent addition of H_2_/D_2_ would then form an alkyl hydride, **A**, followed by reductive bond formation to give **[1-COA][BAr**^**F**^_**4**_**]**. This would result in **d**_**4**_-**[1-COA][BAr**^**F**^_**4**_**]** using D_2_, contrary to the higher levels of deuteration observed. The levels of H/D incorporation in COA closely match those for COE, even at low conversions to COA_TOTAL_, suggesting the exchange occurs principally from **[1-COE][BAr**^**F**^_**4**_**]** and not **[1-COA][BAr**^**F**^_**4**_**]**. However, as H/D exchange has been reported for other well-defined σ-alkane complexes, we cannot rule out that this does not also occur here.^11,18^ H/D exchange at **[1-COE][BAr**^**F**^_**4**_**]** could proceed by two routes: (a) formation of an alkyl deuteride, **A**, with D_2_, which if followed by β-elimination from a different α-methylene group, would return D-labeled **[1-COE][BAr**^**F**^_**4**_**]**; (b) C–H activation of COE to form an allyl-hydride, **B**, H/D exchange with D_2_ at the hydride, and a 1,3-deutride shift to also return D-labeled **[1-COE][BAr^F^_4_]**. Similar intermediates to **B** have been described for the solid-state hydrogenation of [Ir(PPh_3_)_2_(COD)]_3_[PW_12_O_40_].^19^ Both processes would result in double bond isomerization. Repetition exchanges all the C–H bonds for C–D, if combined with a process that allows for all C–H bonds to interact with the Rh center (see below). Consistent with H/D exchange at a Rh–H intermediate that introduces a single D atom, there is no enhancement of odd or even numbers of d_*x*_ (Supporting Information). These reversible processes must be thermodynamically balanced and be connected by low barriers for such rapid H/D exchange to occur. Periodic DFT calculations on hydride insertion/β-elimination show this to be the case for closely related [Rh(Cy_2_PCH_2_CH_2_PCy_2_)(cyclohexene)][BAr^F^_4_] in the solid state with barriers being less than 13.9 kcal mol^–1^.^11^ A low barrier to a 1,3-hydride shift in the isomerization of propene in [Rh(Cy_2_PCH_2_CH_2_PCy_2_)(propene)][BAr^F^_4_] that operates via an allyl hydride intermediate has also been reported (10.9 kcal mol^–1^).^22^ H/D exchange at Rh(III)-H with D_2_ would likely operate via a σ-CAM mechanism.^47,48^

**Scheme 3:**
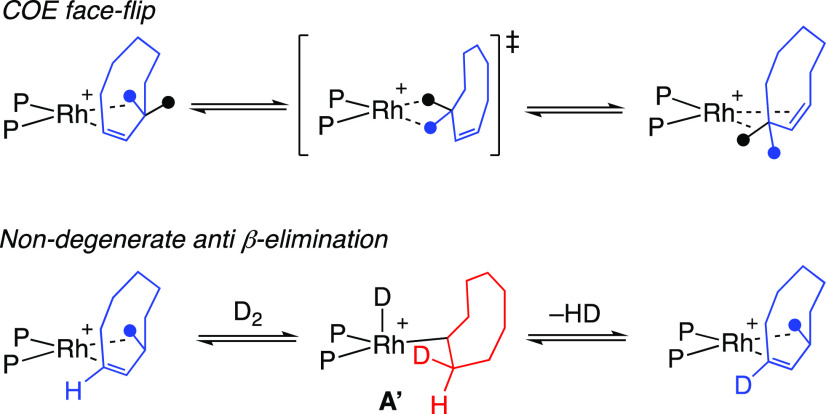
Suggested Pathways That Allow for Perdeuteration

In order to achieve such high levels of deuteration in both COE and COA, all the C–H bonds in the cyclic hydrocarbon need to interact with the metal center, and the pathways shown in Scheme 2 would result in only one face of COA being deuterated. Scheme 3 suggests routes that allow for both faces to be deuterated: a face flip of COE or a nondegenerate β-elimination of an antiorientated C–H bond from intermediate **A′** that may be promoted by ring strain in the cyclooctyl ligand.^49^ Similarly high levels of H/D exchange in the resulting COA have also been reported in solid/gas reactions of [Ir(PPh_3_)_2_(COD)]_3_[PW_12_O_40_] with D_2_.^19^

Hydrogenation in the THF solution of **[1-COD][BAr**^**F**^_**4**_**]** results in the formation of COA-*d*_4_ with no H/D exchange observed. Thus the microenvironment provided by the [BAr^F^_4_]^−^ anions, that prevents rapid loss of the bound COE (and COA) in the solid-state, facilities the extremely high levels of H/D exchange in this SC–SC process.

The rapid deuteration of **[1-COE][BAr**^**F**^_**4**_**]** results in nearly complete H/D exchange of COE. This could lead to an inverse equilibrium isotope effect (EIE) being observed for the consumption of **[1-COE][BAr**^**F**^_**4**_**]**, as deuteration would be expected to bias any equilibria toward **d_*x*_**-**[1-COE][BAr**^**F**^_**4**_**]**, and intermediate **A** (assuming a small, or even inverse,^5^ isotope effect for H_2_ addition to **[1-COE][BAr**^**F**^_**4**_**]**) and away from **B**. While this assumes reductive bond cleavage from **A** to form **[1-COA][BAr**^**F**^_**4**_**]** is rate determining, COA loss from **[1-COA][BAr**^**F**^_**4**_**]** to form **[1][COA⊂BAr**^**F**^_**4**_**]** could also be rate determining, as the solution quenching experiments do not discriminate between these two species in measuring [COA]_TOTAL_ (Figure 4).

This analysis is further complicated by a number of factors that are unique to the solid-state reactivity described here. The cumulative effects of thermodynamically favorable perdeuteriation,^50^ feasible because of the encapsulation, will induce a net secondary isotope effect on the reductive bond formation from **A**. Isotopologue induced changes in noncovalent interactions between the alkane and anion microenvironment will also affect both the equilibrium thermodynamics and transition state energetics and, thus, may also contribute to the observed isotope effects. Related binding isotope effects (BIEs) have been observed with enzymes and molecular capsules on binding different isotopologues of the same guest substrate.^2^ So, while the observation of an inverse isotope effect in a SC–SC molecular organometallic solid/gas reaction is clear-cut here, the additional complexity introduced by reactivity in the single crystal makes the detailed analysis of the underlying reasons for this more challenging.

An inverse isotope effect has been reported for the solution-based deuteration of NBD using [Ir(PPh_3_)_2_H_2_(acetone)_2_][PF_6_] and is explained by a mechanism that favors norborenyl-hydride intermediates closely related to intermediates described here such as **A**.^51^ Inverse EIEs have previously been used to identify the intermediacy of σ-alkane complexes in overall reductive elimination of alkanes from alkyl-hydrides in solution where the loss of the alkane from the metal center is rate determining.^4^

## Conclusions

The study of isotope effects has been central to the understanding of mechanisms in organometallic synthesis and catalysis in the solution phase. The inverse isotope effect described here for the sequential SC–SC hydrogenation of **[1-COD][BAr**^**F**^_**4**_**]** adds to the small number of reports where (albeit normal) isotope effects have been noted in molecular organometallic chemistry in the crystalline phase.^11,16−20^ The leverage of the isotopologue-induced changes in relative rates results in the structural characterization of a σ-alkane complex of cyclooctane. While reactivity in the single-crystalline environment presents challenges in both data collection and analysis of isotope effects, the installed secondary microenvironment around the reactive metal center promotes temporal control over composition, stability (σ-alkane complex formation), and reactivity (extensive H/D exchange). This highlights that the advantages of isotopic substitution in the study of mechanism and synthesis are not unique to homogeneous systems, and it should also be considered as a useful tool in SC–SC transformations of molecular organometallics.

## Supporting Information Available

Full details of synthesis, characterization (including single crystal X-ray determinations), kinetic measurement protocols, sequential JMAK analysis, and connectivity-only structure for the redetermination of **[1][COA⊂BAr**^**F**^_**4**_**]** using D_2_ (PDF)
Computational details, electronic structure analysis, and optimized structure of **[1-COA][BAr**^**F**^_**4**_**]** (XYZ)

## Supplementary Material

om1c00639_si_001.pdf

om1c00639_si_002.xyz

## Accession Codes

CCDC 2120252 contains the supplementary crystallographic data for this paper. These data can be obtained free of charge via www.ccdc.cam.ac.uk/data_request/cif, or by emailing data_request@ccdc.cam.ac.uk, or by contacting The Cambridge Crystallographic Data Centre, 12 Union Road, Cambridge CB2 1EZ, UK; fax: +44 1223 336033.
