# Supplementary material for: Inverse Isotope Effects in Single-Crystal to Single-Crystal Reactivity and the Isolation of a Rhodium Cyclooctane σ-Alkane Complex
Source: Organometallics. 2022 Jan 27;41(3):284–92. doi: 10.1021/acs.organomet.1c00639 (PMC8900153; doi:10.1021/acs.organomet.1c00639)
Supplement: Supplementary file 1 — om1c00639_si_001.pdf [file om1c00639_si_001.pdf]

Supporting Information

Inverse Isotope Effects in Single-Crystal to Single-Crystal Reactivity and the Isolation of a Rhodium Cyclooctane σ-Alkane Complex.

Laurence R. Doyle,*[a] Martin R. Galpin,[b] Samantha K. Furfari,[a] Bengt E. Tegner,[c] Antonio J. Martínez-Martínez,[d] ‡ Adrian C. Whitwood,[a] Scott A. Hicks,[a] Guy C. Lloyd-Jones,[e] Stuart A. Macgregor,*[c] and Andrew S. Weller*[a]

[a] Department of Chemistry, University of York, Heslington, York, YO10 5DD (UK)

[b] Physical and Theoretical Chemistry Laboratories, University of Oxford, Oxford, OX1 3QZ (UK)

[c] School of Chemical Sciences, Heriot-Watt University, Edinburgh, Scotland, EH14, 4AS (UK)

[d] Department of Chemistry, Mansfield Road, University of Oxford, Oxford, OX1 3TA (UK)

[e] Department of Chemistry, University of Edinburgh, Edinburgh, Scotland, EH9 3FJ (UK).

‡ Present address: Centro de Investigación en Química Sostenible (CIQSO), Edificio Robert H Grubbs, Campus de El Carmen, Universidad de Huelva 21007 Huelva (Spain)

S.1 EXPERIMENTAL DETAILS **S3**

S.2 SYNTHETIC PROCEDURES **S3**

S.2.1 Solid-state hydrogenation of **[1-COD][BAr$^F_4$]**: in-situ solid-state NMR experiments

S.2.2 Solid-state hydrogenation of **[1-COD][BAr$^F_4$]**: solution quenching experiments

S.2.3 **[1-COA][BAr$^F_4$]** quenching with acetone-d$_6$: in situ formation of [Rh(Cy$_2$P(CH$_2$)$_3$PCy$_2$)(acetone-d$_6$)$_2$][BAr$^F_4$]

S.2.4 **[1-COA][BAr$^F_4$]** quenching with MeCN-d$_3$: in situ formation of [Rh(Cy$_2$P(CH$_2$)$_3$PCy$_2$)(MeCN-d$_3$)$_2$][BAr$^F_4$]

S.2.5 Solid-state synthesis of **[1-H$_x$][BAr$^F_4$]** and **[1-D$_x$][BAr$^F_4$]** and dissolution in MeCN-d$_3$.

S.2.6 Solution hydrogenation of **[1-COD][BAr$^F_4$]** with D$_2$

S.3 CRYSTALLOGRAPHIC AND REFINEMENT DATA **S12**

S.3.1 X-ray crystal structure of **[1-COA][BAr$^F_4$]**

S.3.2 Unit cell and partial X-ray structural solution of **[1][COA⊂BAr$^F_4$]**

S.4 JOHNSON–MEHL–AVRAMI–KOLMOGOROV (JMAK) ANALYSIS **S17**

S.4.1 Derivation of the rate equations for the sequential process

S.4.2 Numerical solution of the differential equations

S.5 COMPUTATIONAL METHODS **S22**

S.5.1 QTAIM study of **[1-COA]$^+$**

3.5.2 Non-covalent interaction (NCI) study of the **[1-COA][BArF$_4$]** ion-pair

S.5.3 Natural bond orbital analysis of the **[1-COA]$^+$** cation

S.5.4 Computed energy for **[1-COA][BAr$^F_4$]**

S.6 REFERENCES **S27**

S.1 EXPERIMENTAL DETAILS

All manipulations (unless stated otherwise) were performed under an argon atmosphere, using standard Schlenk techniques on a dual vacuum/argon manifold or by using an argon filled glovebox (MBraun). Glassware was flame dried under vacuum prior to use. Pentane and dichloromethane (CH_2Cl_2) were dried using an Innovative Technology Pure-Solv™ (PS-400-3) solvent purification system and degassed by freeze-pump-thaw cycles. Deuterated solvents were dried using an appropriate drying agent: dichloromethane-d_2 (CD_2Cl_2) with CaH_2; acetonitrile-d_3 (MeCN-d_3) with 3 Å molecular sieves; and acetone-d_6 with B_2O_3. After drying, these solvents were degassed by freeze-pump-thaw cycles and then stored over 3 Å molecular sieves (except acetone-d_6). Hydrogen (H_2) and deuterium (D_2) gases were purchased in lecture bottles from Sigma-Aldrich and used as received. **[1-COD][BAr$^F_4$]** was prepared by the literature procedure.[1] All other chemicals were purchased from commercial vendors and used as received.

Solution NMR data were collected on either a Bruker AVIIIHD 500 MHz or 600 MHz spectrometer at 298 K unless otherwise stated. Residual protio solvent resonances were used as a reference for ^{1}H NMR spectra.[2] ^{31}P{^{1}H} NMR spectra were referenced externally to 85 % H_3PO_4 (D_2O). All chemical shifts (δ) are quoted in ppm and coupling constants in Hz.

Solid-state NMR samples were prepared by packing powdered microcrystalline samples into a 4 mm zirconia rotor inside an argon filled glove box. ^{31}P{^{1}H} solid-state NMR spectra were obtained on a Bruker AVIIIHD 400 spectrometer, with a magic-angle spinning (MAS) rate of 10 kHz, referenced to triphenylphosphine (δ = –9.3).[3]

Gas chromatographic analysis was carried out using a Thermo Trace 1300 GC equipped with an AI 1310 autosampler. Separation was achieved using a Rxi-17 column (30 m × 0.25 mm, 0.25 μm film thickness) with a carrier gas (H_2) flow rate of 2 mL min^{-1} and a temperature ramp from 40 to 120 °C at 3 °C min^{-1}. The injection volume was 1 μL with a split ratio of 3.

MS spectra were measured using a JEOL AccuTOF GCx-plus instrument (JMS-T200GC) using electrospray ionisation.

S.2 SYNTHETIC PROCEDURES

S.2.1 Solid-state hydrogenation of [1-COD][BAr$^F_4$]: in-situ solid-state NMR experiments

[1-COD][BAr$^F_4$] (~50 mg, ~0.033 mmol) was packed within a solid-state NMR rotor and measured by ^{31}P solid-state NMR. For each successive hydrogenation period, the rotor was

uncapped within an Ar glovebox, then placed within a sealed ampoule (~10 cm^3). After evacuating the ampoule to 0.01 mbar pressure, the headspace was filled with either H_2 or D_2 (1.5 bar) for a set time, after which the ampoule was briefly evacuated to 0.01 mbar pressure, then backfilled with Ar (1.2 bar). After recapping the rotor in an Ar glovebox (1 bar), the sample was remeasured by ^{31}P solid-state NMR.

S.2.2 Solid-state hydrogenation of [1-COD][BAr$^F_4$]: solution quenching experiments

In separate experiments, ground crystals of **[1-COD][BAr$^F_4$]** (7.6 mg, 0.005 mmol, ca 71-150 μm diameter) were hydrogenated for a set time (0-300 min) under an atmosphere of either H_2 or D_2 (1.5 bar, ~ 30 eq.) in a sealed NMR tube (~ 2.5 cm^3). The solids were then frozen by submersion of the tube in a liquid nitrogen bath and degassed by evacuation of the tube to 0.01 mbar. After dissolution in acetone-d_6 (0.5 mL), the resultant mixture, containing **[1-COD][BAr$^F_4$]**, [Rh($Cy_2P(CH_2)_3PCy_2$)(acetone-d_6)$_2$][BAr$^F_4$], free COE and COA, was quantified by ^1H NMR spectroscopy. MeCN (50 μL, 189 eq.) was subsequently added to the mixture to convert all Rh-species to [Rh($Cy_2P(CH_2)_3PCy_2$)(MeCN)$_2$][BAr$^F_4$] and liberate free COD. The volatiles, now containing free COD, COE and COA, were collectively distilled at 0.01 mbar pressure and subsequently quantified by GC; in the experiments with D_2 gas, the degree of deuteration in COD, COE, and COA species was determined by GC-MS.

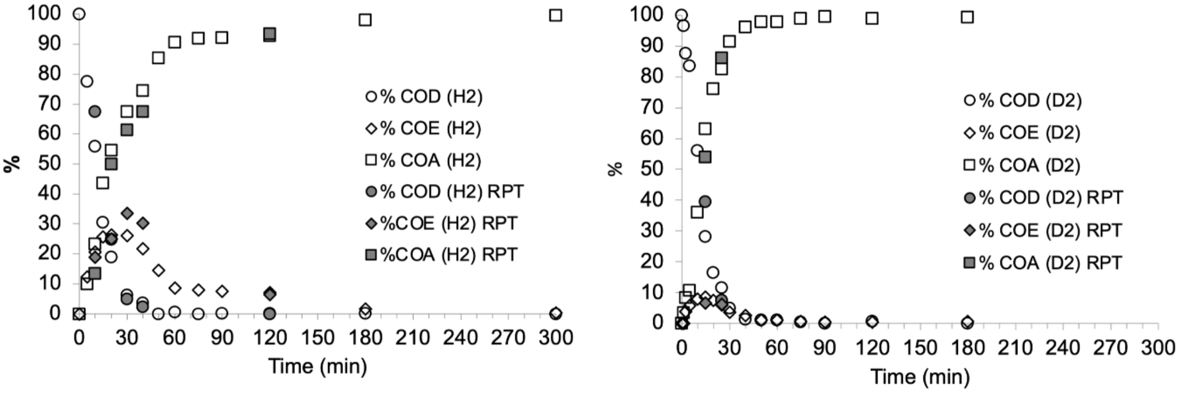

Figure S1. Time/conversion plots as measured by GC/MS of quenched samples for addition of H_2 (left) or D_2 (right) to **[1-COD][BAr$^F_4$]** using sieved (71 μm to 150 μm) crystalline sample (1.5 bar gas, 298 K). Each data point is a different experiment. Open markers = data reported in main text. Filled markers = selected repeat experiments using a different batch of sample.

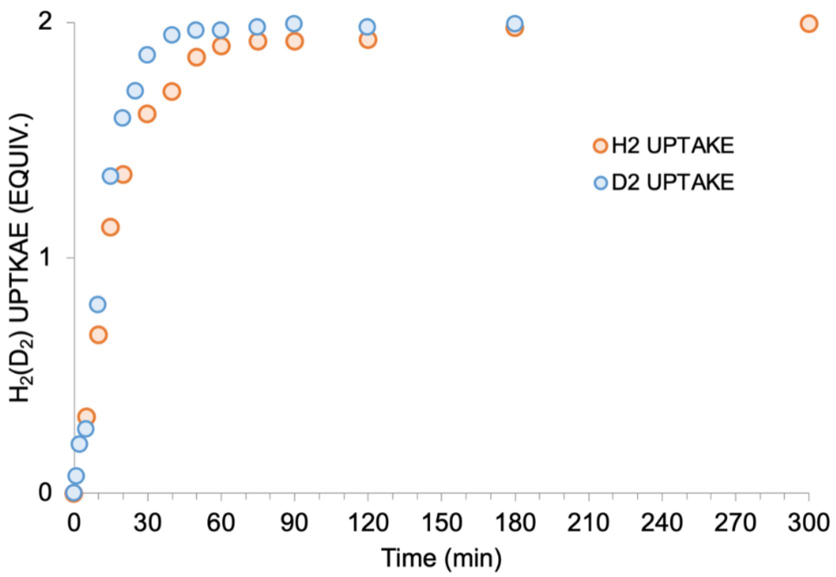

Figure S2. Time versus calculated H_2 or D_2 uptake plot for addition of H_2 to D_2 to **[1-COD][BArF4]**. Note the very similar temporal profile for the early stages of the reaction (hydrogenation of **[1-COD][BArF4]**) with D_2 uptake being faster for the later stages (formation of **[1-COA][BArF4]**). Combined these observations suggest the reaction is not diffusion limited.

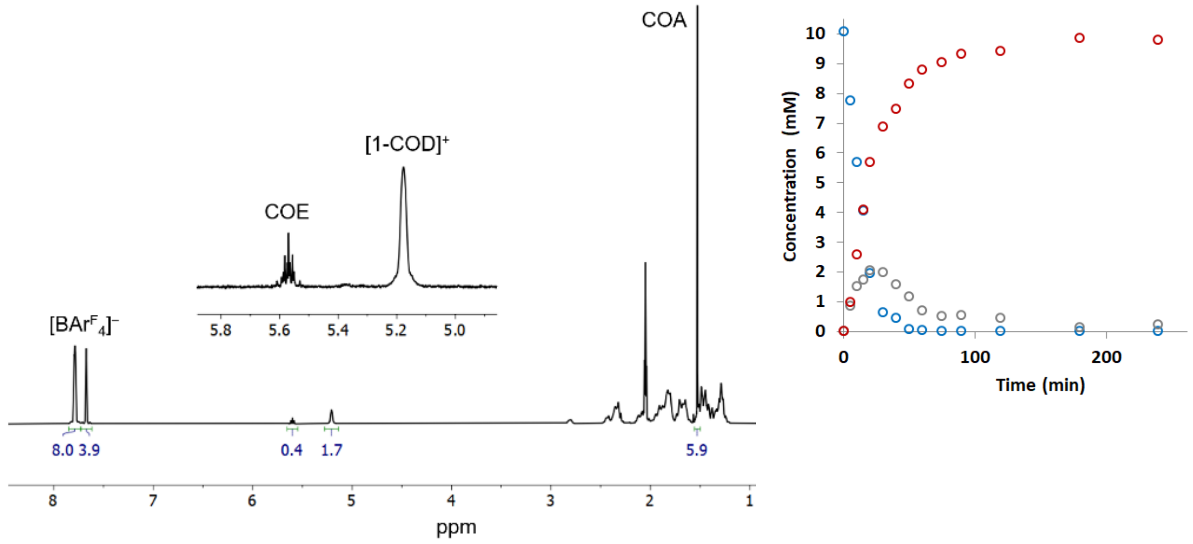

Figure S3. ^{1}H NMR quantification of the solid-gas reactions of **[1-COD][BAr^{F}4]** with H_2. Left: exemplar ^{1}H NMR (acetone-d_6) spectrum after 15 min hydrogenation. Right: temporal evolution of **[1-COD][BAr^{F}4]**, COE and COA concentrations in acetone-d_6; each data point represents a separate experiment.

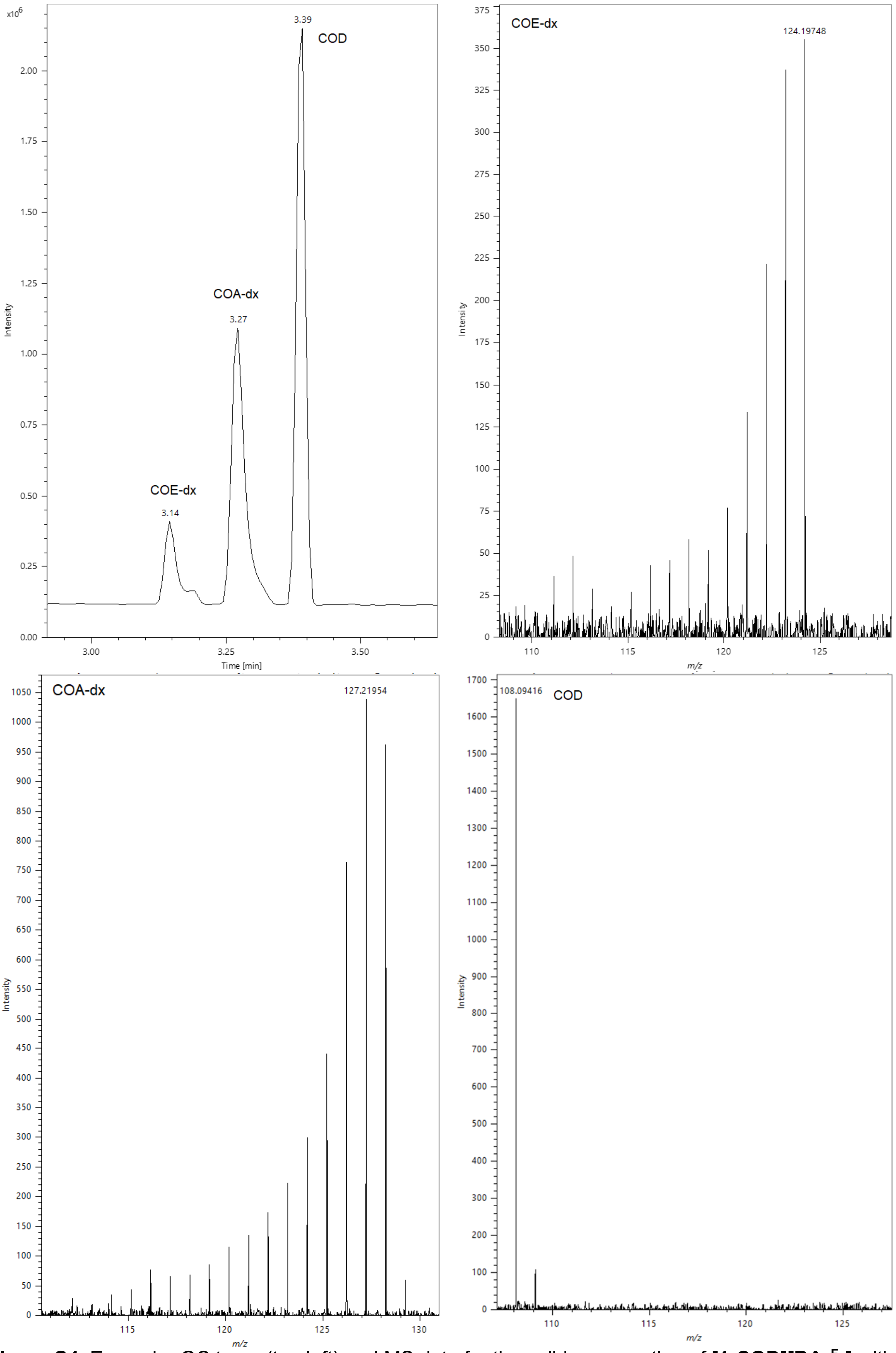

Figure S4. Exemplar GC trace (top left) and MS data for the solid-gas reaction of **[1-COD][BAr^F$_4$]** with D_2 (1.5 bar, 10 min).

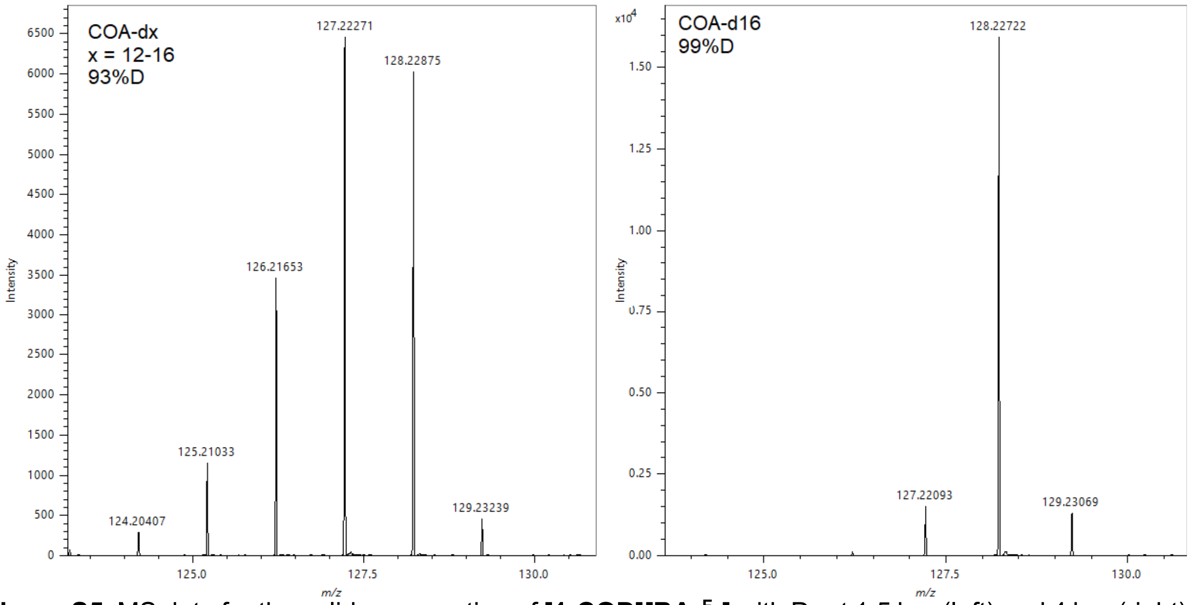

Figure S5. MS data for the solid-gas reaction of **[1-COD][BAr^F$_4$]** with D_2 at 1.5 bar (left) and 4 bar (right) for 180 min.

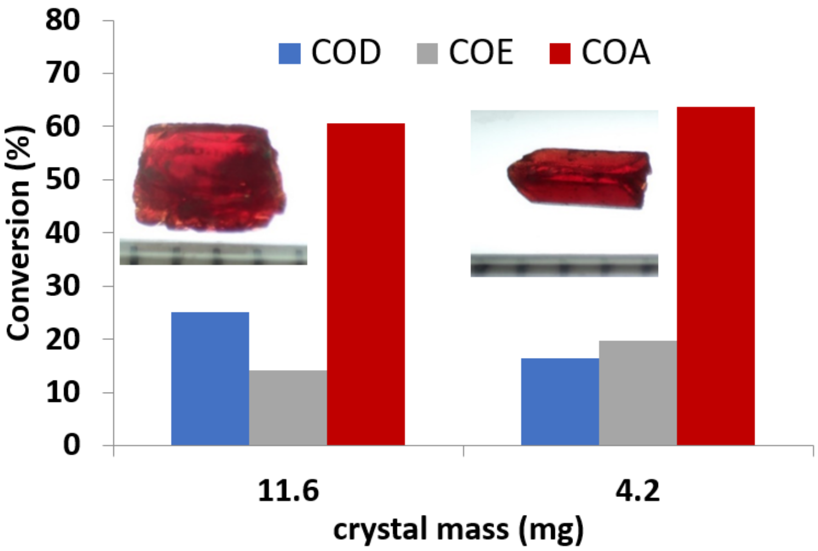

Figure S6. Comparison between crystal sizes for the solid-gas reaction of **[1-COD][BAr^F$_4$]** with H_2 at 1.5 bar for 50 min; the insets show the respective crystals alongside a 4mm scale.

S.2.3 [1-COA][BAr^F$_4$] quenching with acetone-d_6: in situ formation of [Rh($Cy_2P(CH_2)_3PCy_2$)(acetone-d_6)$_2$][BAr^F$_4$]

[1-COD][BAr^F$_4$] (7.6 mg, 0.005 mmol) was hydrogenated with D_2 (1.5 bar) for 60 min in a sealed NMR tube (~ 2.5 cm^3). The solids were then frozen by submersion of the tube in a liquid nitrogen bath and degassed by evacuation of the tube to 0.01 mbar. After warming to 298 K, the solids were dissolved in acetone-d_6 (0.5 mL).

^{1}H NMR (acetone-d$_6$, 298 K, 400 MHz): δ 7.79 (br m, 8H, ortho-ArH), 7.68 (s, 4H, para-ArH), 2.39-2.29 (br m, 4H, P-CH$_2$CH$_2$-P), 1.98-1.59 (br m, 24H, aliphatic CH), 1.56-1.39 (br m, 8H, aliphatic CH), 1.39-1.20 (br m, 12H, aliphatic CH).

^{31}P{^{1}H} NMR (acetone-d$_6$, 298 K, 162 MHz): δ 45.78 (d, J_{RhP} = 193 Hz).

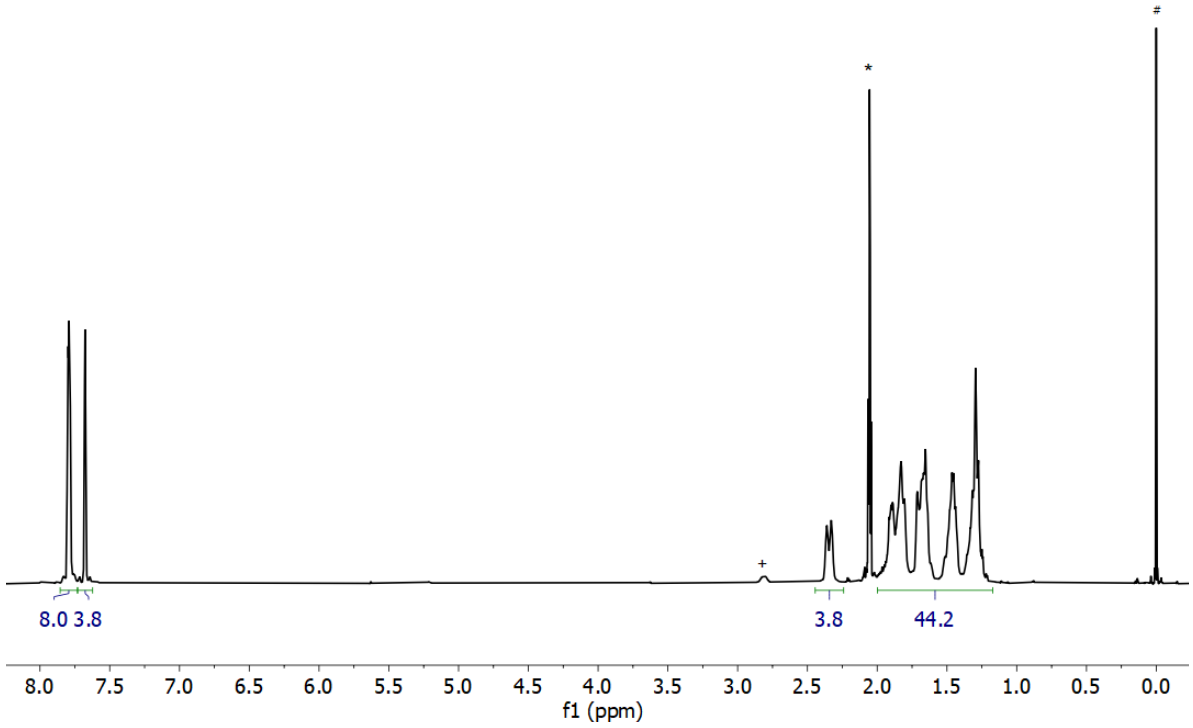

Figure S7. ^{1}H NMR spectrum of [Rh(Cy$_2$P(CH$_2$)$_3$PCy$_2$)(acetone-d$_6$)$_2$][BAr$^F{}_4$] in acetone-d$_6$ (*); # = SiMe$_4$, + = H$_2$O.

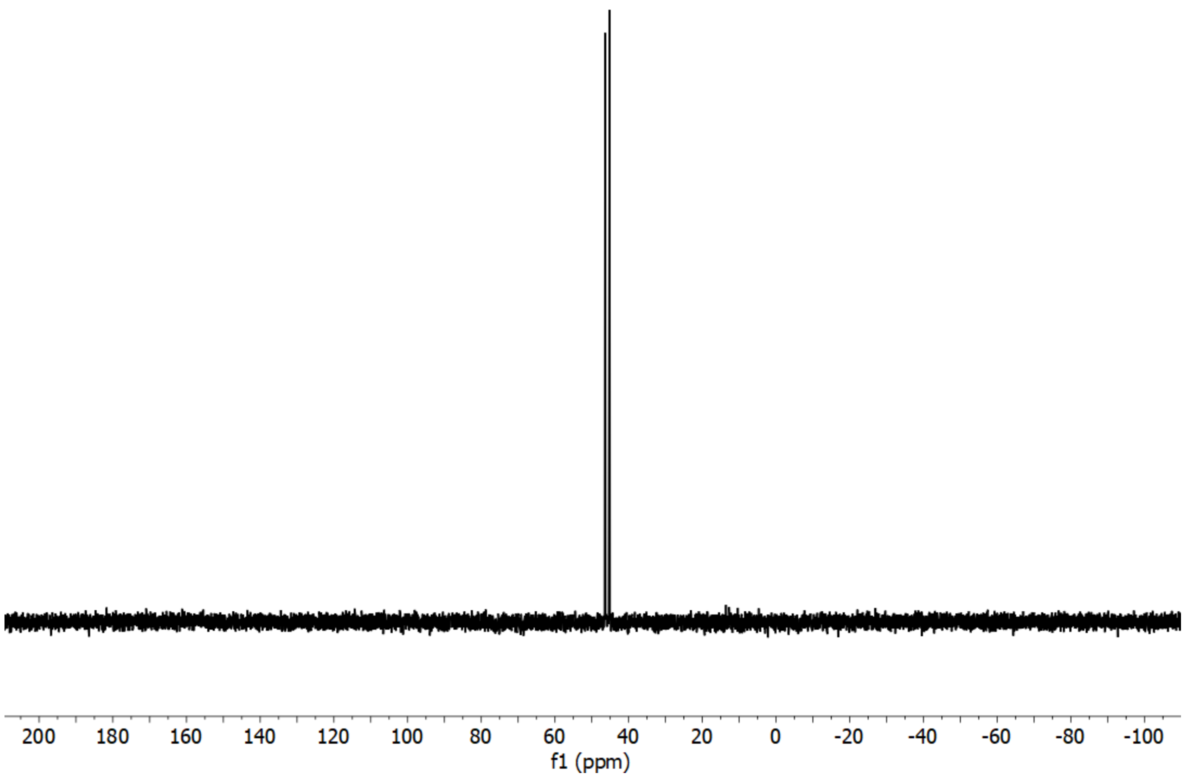

Figure S8. $^{31}P\{^1H\}$ NMR spectrum of [Rh($Cy_2P(CH_2)_3PCy_2$)(acetone-d_6)$_2$][BAr^F_4] in acetone-d_6.

S.2.4 [1-COA][BAr^F_4] quenching with MeCN-d_3: in situ formation of [Rh($Cy_2P(CH_2)_3PCy_2$)(MeCN-d_3)$_2$][BAr^F_4]

[1-COD][BAr^F_4] (7.6 mg, 0.005 mmol) was hydrogenated with H_2 (1.5 bar) for 17h in a sealed NMR tube (~ 2.5 cm^3). The solids were then frozen by submersion of the tube in a liquid nitrogen bath and degassed by evacuation of the tube to 0.01 mbar. After warming to 298 K, the solids were dissolved in MeCN-d_3 (0.5 mL).

^1H NMR (MeCN-d_3, 298 K, 400 MHz): δ 7.69 (br m, 8H, ortho-ArH), 7.67 (s, 4H, para-ArH), 2.32-2.22 (br m, 4H, P-CH_2CH_2-P), 1.89-1.65 (br m, ~22H, aliphatic CH), 1.45-1.18 (br m, ~25H, aliphatic CH).

$^{31}P\{^1H\}$ NMR (MeCN-d_3, 298 K, 162 MHz): δ 36.11 (d, J_{RhP} = 167 Hz).

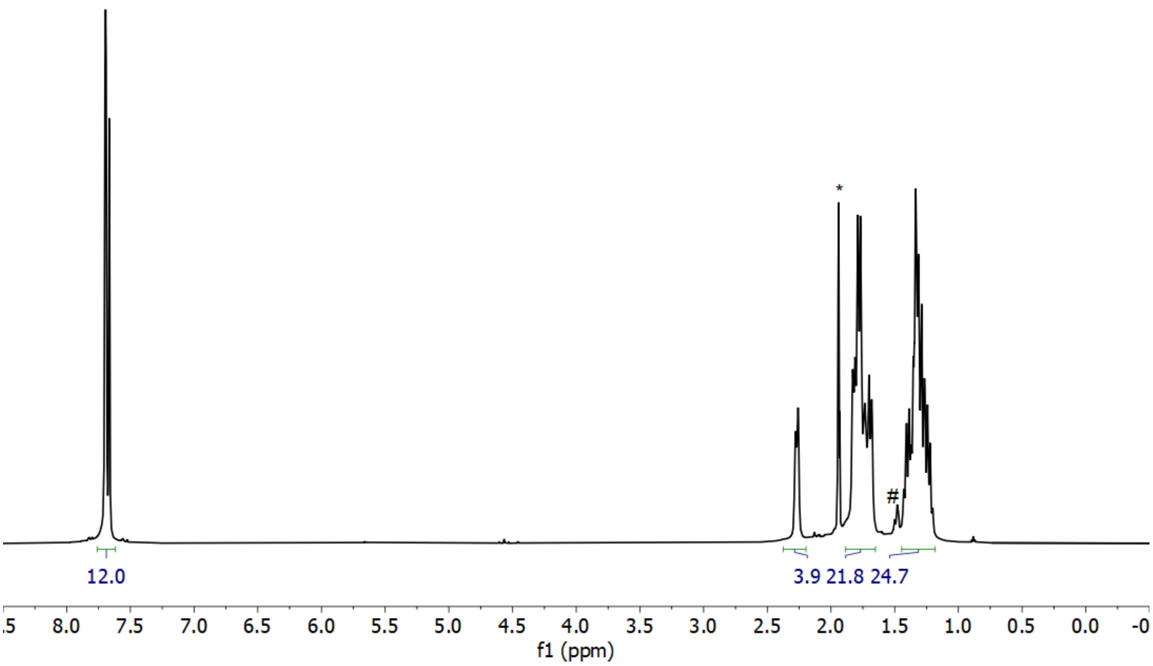

Figure S9. ^{1}H NMR spectrum of [Rh($Cy_2P(CH_2)_3PCy_2$)(MeCN-d_3)$_2$][$BAr^F{}_4$] in MeCN-d_3 (*); # = COA-d_x.

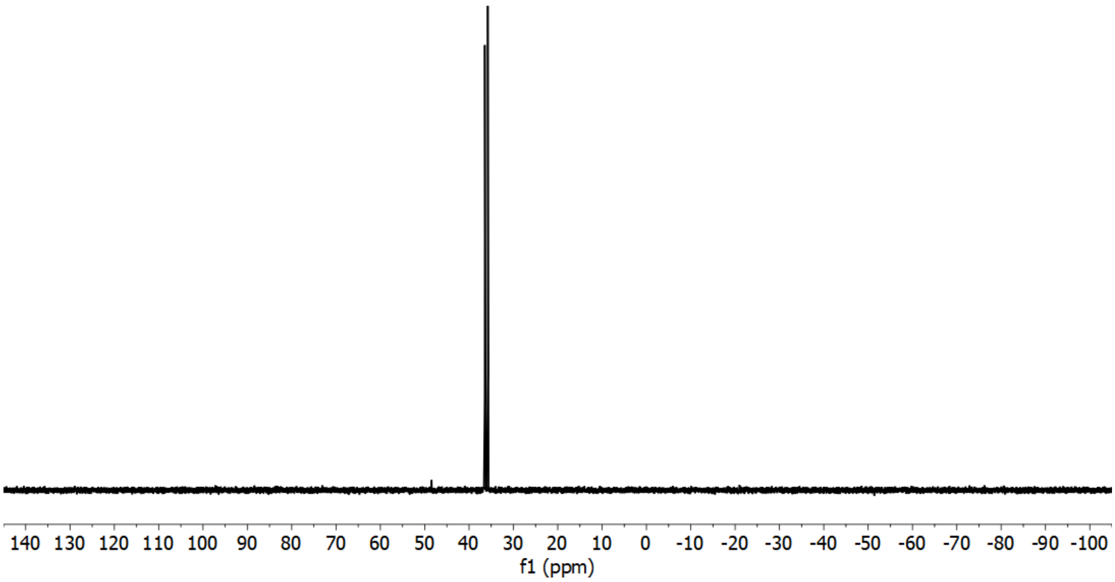

Figure S10. $^{31}P\{^{1}H\}$ NMR spectrum of [Rh($Cy_2P(CH_2)_3PCy_2$)(MeCN-d_3)$_2$][$BAr^F{}_4$] in MeCN-d_3.

S.2.5 Solid-state synthesis of [1-H_x][$BAr^F{}_4$] and [1-D_x][$BAr^F{}_4$] and dissolution in MeCN-d_3.

[1-COD][$BAr^F{}_4$] (7.6 mg, 0.005 mmol) was hydrogenated with either H_2 or D_2 (1.5 bar) for 17h in a sealed NMR tube (~ 2.5 cm^3), then degassed by three evacuation (0.01 mbar)/argon refill cycles to remove H_2 or D_2 in the headspace. The remaining solid was frozen in a liquid nitrogen bath and then MeCN-d_3 (0.5 mL) was added via vacuum distillation. After removing the liquid nitrogen bath, the headspace was refilled with Ar (1.2 bar).

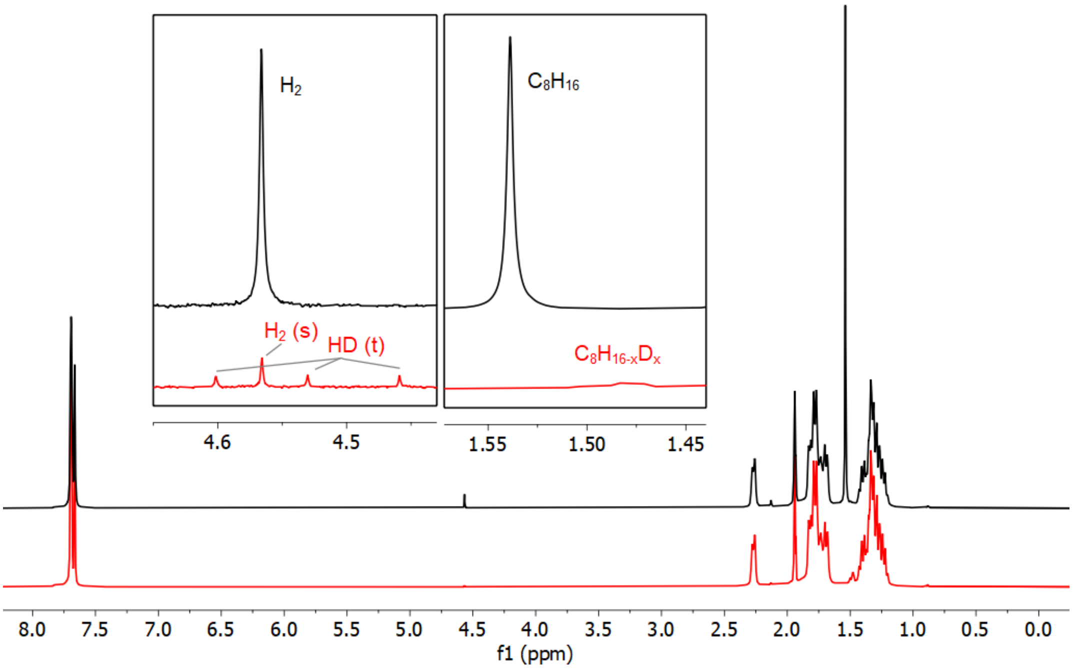

Figure S11. ^{1}H NMR spectra of **[1-H$_x$][BAr$^F_4$]** (black) and **[1-D$_x$][BAr$^F_4$]** (red) after dissolution in MeCN-d_3 and liberation of free $(H/D)_2$.

S.2.6 Solution hydrogenation of [1-COD][BAr$^F_4$] with D_2

In a ca 15 cm^3 Rotaflo® ampoule containing a magnetic stirrer bar and an unused rubber septum, a THF (5 mL) solution of **[1-COD][BAr$^F_4$]** (37.8 mg, 0.025 mmol, 5 mM) was freeze-pump-thaw degassed three times, then placed under a dynamic atmosphere of D_2 (1.33 bar) with fast stirring at 500 rpm. 0.5 mL aliquots were removed after 2 min and 60 min via cannula transfer into Ar-filled vials containing MeCN (53 µL, 1 mmol, 40 eq.) to convert all Rh-species to [Rh(dcpp)(MeCN)$_2$][BAr$^F_4$], exclusively. The volatiles, now containing free COD, COE and COA, were collectively distilled at 0.01 mbar pressure and subsequently quantified by GC-MS. In both aliquots, the degree of deuteration in COD, COE, and COA species was determined by GC-MS to be: COD-d_0, COE-d_2, and COA-d_4.

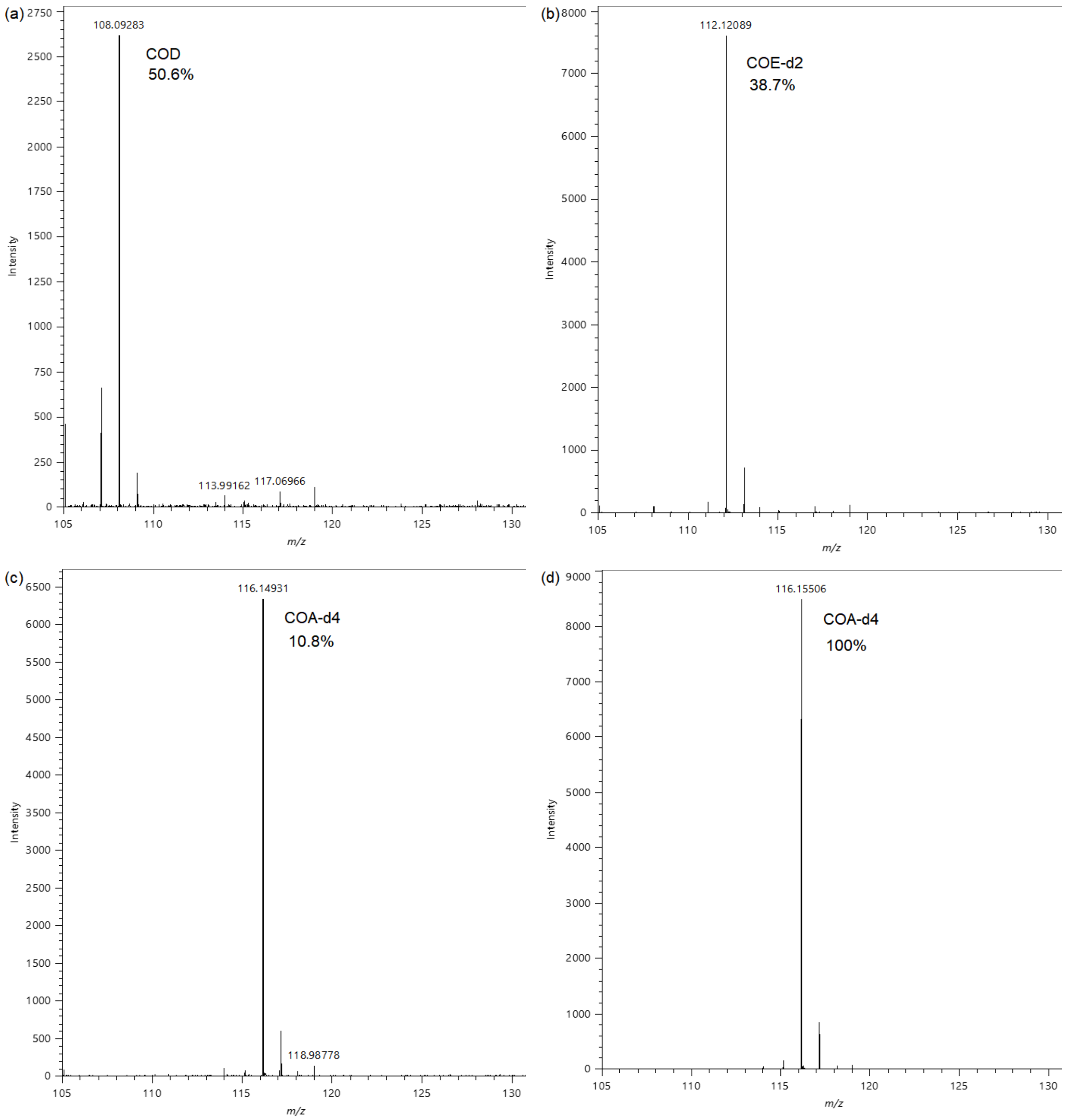

Figure S12. Mass spectra for the COD, COE, and COA fractions (relative quantities shown as percentages) from the solution D_2-hydrogenation of **[1-COD][BAr^F$_4$]** after (a-c) 1 min and (d) 60 min.

S.3 CRYSTALLOGRAPHIC AND REFINEMENT DATA

Single-crystal X-ray diffraction data for **[1-COA][BAr^F$_4$]** and **[1][COA⊂BAr^F$_4$]**[1] were collected on an Oxford Diffraction SuperNova diffractometer with Cu-*Kα* (λ = 1.54184 Å) radiation equipped with a nitrogen gas Oxford Instruments Cryojet cooler. Raw frame data was reduced using CrysAlisPro, solved using Superflip[4], and refined using full-matrix least squares refinement on all F^2 data using SHELXL-18[5] within the OLEX2 program.[6] All non-hydrogen atoms were refined anisotropically and hydrogen atoms were geometrically placed unless otherwise stated and allowed to ride on their parent atoms. Distances and angles were

calculated using the full covariance matrix. Selected crystallographic data are summarized in the text and full details are given in the supplementary deposited CIF file (CCDC: 2120252). This data can be obtained free of charge from the Cambridge Crystallographic Data Centre via http://www.ccdc.cam.ac.uk/data_request/cif.

S.3.1 X-ray Crystal Structure of [1-COA][BAr$^F{}_4$]

The crystal was found to be a pseudo-merohedral twin with two twin components in a refined ratio of 0.5317(14):0.4683(14) and twin law: 0 -1 0 -1 0 0 0 0 -1. The structure is modelled as a mixture of three species, which share common atom positions within the anion, but have some differences in the atom positions within the cation, notably Rh1, Rh2 and Rh3; the occupancies of the three species were allowed to freely refine, converging at a final ratio of 65.5(5):22.2(6):12.3(4), respectively. In the major component, a cyclooctane molecule bound to Rh1 was modelled with an occupancy of 1; this incorporates the electron density from the corresponding C8-hydrocarbon molecule in the two minor components, which could not be located separately in the electron density map, and is reflected in the relatively large ADPs. If no atoms are modelled in the pocket in which the cyclooctane molecule resides, a PLATON-SQUEEZE[7] calculation estimates a pocket volume of 195 Å^3 containing approximately 67 electrons; the modelled cyclooctane molecule, which contains 64 electrons, has a calculated volume of 115 Å^3 (OLEX2: r_w(C) = 1.7; r_w(H) = 1.09). The diphosphine ligand occupies two orientations with respect to the P atoms. Rh1 and Rh2 are bonded to the major orientation (87.7%) centred on P1 and P2, whilst Rh3 is bonded to the minor orientation centred on P1B and P2B (12.3%). In the minor orientation of the diphosphine ligand, the carbon positions within the cyclohexyl groups could not be located, and hence the residual electron density (0.74 electrons per carbon) has been accounted for by incorporation into the cyclohexyl groups within the major orientation. Three of these four cyclohexyl groups were found to be disordered over two positions, with freely refined occupancies that converged on final ratios of: 54.2(9):45.8(9), 65.3(10):34.7(10), and 62.5(11):37.5(11). Within the major orientation of the diphosphine ligand, the $P(CH_2)_3P$ backbone is disordered over two positions, for which the freely refined occupancies converged on 0.494(2) and 0.383(2). Within the anion, two CF_3 groups centred on C42 and C59, were found to be disordered over two positions, with freely refined occupancies that converged on final ratios of: 63(2):37(2) and 71(1):29(1), respectively. To aid refinement, similarity anisotropic displacement parameter restraints were applied within the cyclooctane molecule and all disordered components. All chemically equivalent 1,2 Rh-P, 1,2 P-C, and 1,2 C-F distances within the molecule, and all 1,2 C-C

distances within the disordered $P(CH_2)_3P$ backbone were restrained to be approximately equal.

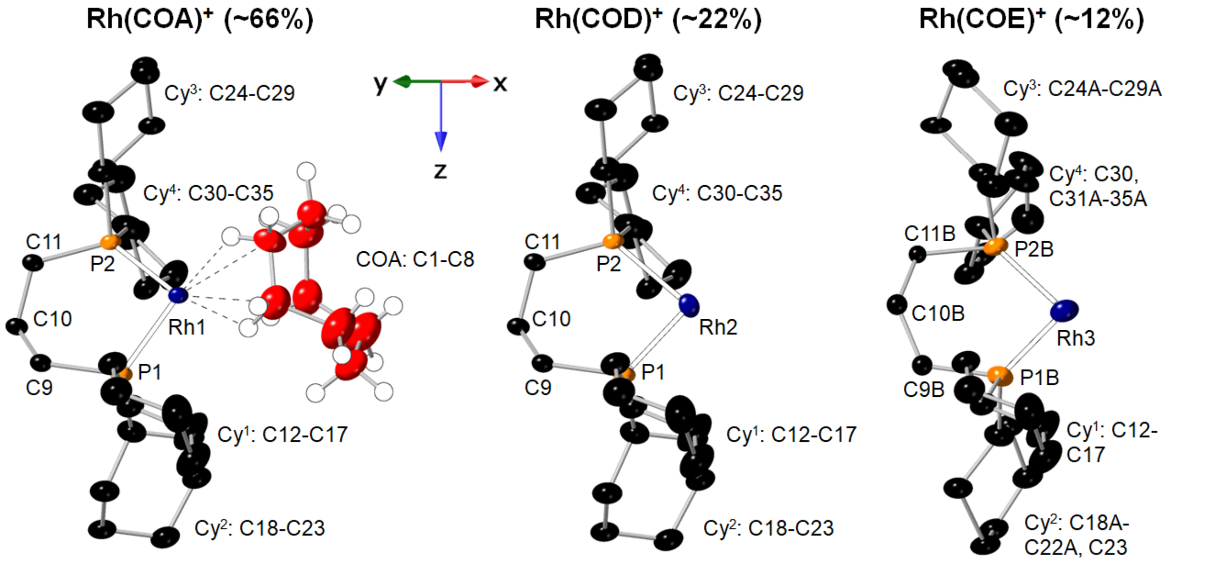

Rh(COA)⁺ and Rh(COD)⁺: *major P only*
major $(CH_2)_3$ backbone only
both major (shown) and minor Cy groups

Rh(COE)⁺: *minor P only*
minor $(CH_2)_3$ backbone only
minor Cy groups only

Part occupancies (%):

Rh1 : Rh2 : Rh3	65.5 : 22.2 : 12.3	Cy1: C12-C17	100
P1/P2 : P1B/P2B	87.7 : 12.3	Cy2: C18-C22 : C18A-C22A	54.2 : 45.8
		C23	100
		Cy3: C24-C29 : C24A-C29A	65.3 : 34.7
		Cy4: C30	100
		C31-C35 : C31A-C35A	62.5 : 37.5

Average Rh-P distances:
Rh1-P 2.209(3) Rh2-P 2.294(6) Rh3-P 2.21(2)

Figure S13. Separated disordered components in the structural solution of **[1-COA][BAr^F$_4$]**. The major component centred on Rh1 is the COA complex, whilst those centred on Rh2 and Rh3 are proposed to be unreactive **[1-COD][BAr^F$_4$]**[1] and intermediate **[1-COE][BAr^F$_4$]**, respectively, based on their Rh-P bond distances and the temporal solid-state NMR of the reaction of **[1-COD][BAr^F$_4$]** with D_2. Only the COA fragment could be located in the electron density map, and was thus modelled at 100% occupancy to incorporate diffuse electron density from unmodelled COE and COD fragments.

Table S1: Selected crystallographic and refinement data for [1-COA][BAr^F$_4$].

Empirical formula	$C_{67}H_{78}BF_{24}P_2Rh$
Formula weight	1514.95
Temperature/K	110(2)
Crystal system	triclinic
Space group	P-1
a/Å	13.1003(14)

b/Å	13.1242(15)
c/Å	20.0837(8)
α/°	87.758(6)
β/°	87.898(5)
γ/°	86.638(9)
Volume/Å^3	3442.4(5)
Z	2
ρ_{calc}g/cm^3	1.462
μ/mm^{-1}	3.377
F(000)	1552.0
Crystal size/mm^3	0.233 × 0.12 × 0.056
Radiation	Cu Kα (λ = 1.54184)
2Θ range for data collection/°	7.946 to 134.152
Index ranges	-15 ≤ h ≤ 14, -15 ≤ k ≤ 14, -23 ≤ l ≤ 23
Reflections collected	25814
Independent reflections	12249 [R_{int} = 0.0569, R_{sigma} = 0.0735]
Data/restraints/parameters	12249/1304/1138
Goodness-of-fit on F^2	1.057
Final R indexes [I>=2σ (I)]	R_1 = 0.0715, wR_2 = 0.1803
Final R indexes [all data]	R_1 = 0.0961, wR_2 = 0.1997
Largest diff. peak/hole / e Å^{-3}	0.84/-0.96
CCDC no.	2120252

S.3.2 Unit cell and partial X-ray structural solution of [1][COA⊂BAr$^F_4$]

Unit cell: Monoclinic, $P2_1/n$, a = 18.9066(14) Å, b = 18.8098(9) Å, c = 19.0074(7) Å, α = 90°, β = 91.945(5)°, γ = 90°, V = 6755.7(6) Å^3, Z = 4.

The solution and refinement of the data collected for **[1][COA⊂BArF4]** is poor (R_1=13.29%) due to low data quality, however the unit cell and connectivity within the major component (73%) matches that reported previously utilising synchrotron radiation (Diamond Light Source,

Beamline I19).[1] Rh1 and Rh2 occupancies were freely refined to sum to 1, resulting in final occupancies of 0.726(3) and 0.274(3), respectively. The P-(CH_2)$_3$-P fragment was refined at an occupancy of 1, assuming bonding to both Rh1 and Rh2. Only the cyclohexyl groups associated with Rh1 could be satisfactorily modelled due to disorder. The encapsulated COA molecule was modelled with two orientations, with freely refined occupancies of 0.56(3) and 0.44(3).

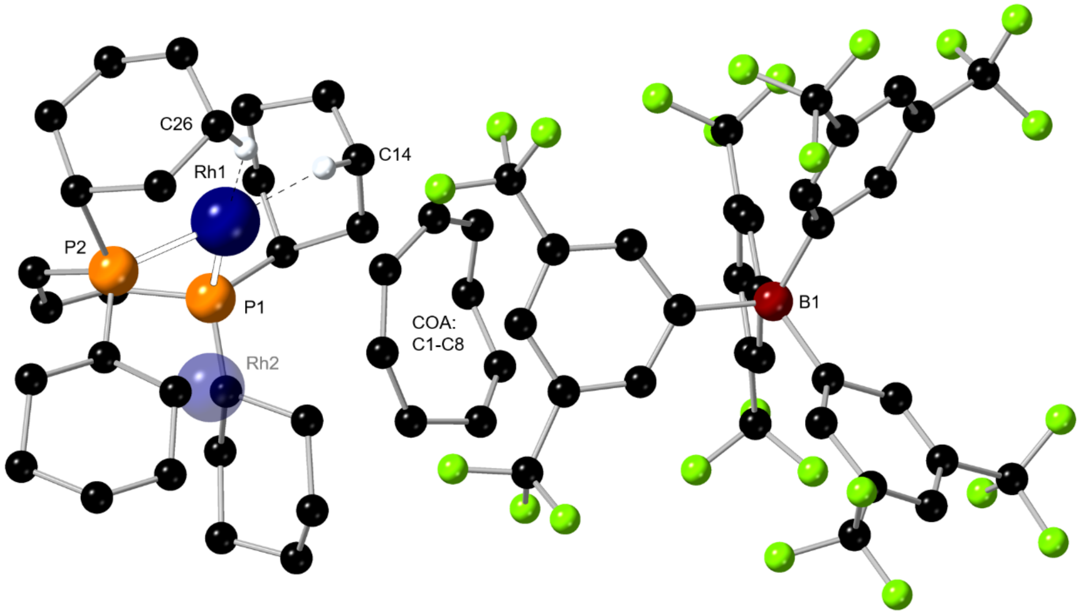

FIGURE S14. Partial structural solution of **[1][COA⊂BArF4]**. H atoms omitted except for the two forming an agostic bond with Rh1. Only the major COA orientation is shown.

Table S2: Selected crystallographic and refinement data for [1][COA⊂BArF4].

Empirical formula	$C_{67}H_{78}BF_{24}P_2Rh$
Formula weight	1514.95
Temperature/K	150(2)
Crystal system	monoclinic
Space group	$P2_1/n$
a/Å	18.9066(14)
b/Å	18.8098(9)
c/Å	19.0074(7)
α/°	90
β/°	91.945(5)

γ/°	90
Volume/Å^3	6755.7(6)
Z	4
ρ_{calc}g/cm^3	1.489
μ/mm^{-1}	3.442
F(000)	3104.0
Crystal size/mm^3	0.137 × 0.106 × 0.096
Radiation	Cu Kα (λ = 1.54184)
2Θ range for data collection/°	8.01 to 100.87
Index ranges	-18 ≤ h ≤ 18, -18 ≤ k ≤ 18, -14 ≤ l ≤ 19
Reflections collected	29815
Independent reflections	7060 [R_{int} = 0.1263, R_{sigma} = 0.0880]
Data/restraints/parameters	7060/1008/939
Goodness-of-fit on F^2	1.296
Final R indexes [I>=2σ (I)]	R_1 = 0.1329, wR_2 = 0.3434
Final R indexes [all data]	R_1 = 0.2108, wR_2 = 0.4059
Largest diff. peak/hole / e Å^{-3}	1.75/-0.73

S.4 JOHNSON–MEHL–AVRAMI–KOLMOGOROV (JMAK) ANALYSIS

S.4.1 Derivation of the rate equations for the sequential process

We consider the sequential reaction

$$A \rightarrow B \rightarrow C$$

starting from pure A at time $t = 0$. We shall assume that $A \rightarrow B$ follows standard Avrami kinetics. The differential equations for this process can be derived by a simple argument. First we consider the 'unrestricted' decrease in the volume of A, between times t and $t + dt$, if there is no B or C already present to hinder further formation of B:

$$dV_A^u = -\, k_B t^3 V \, dt \tag{1}$$

In this expression, V is the total volume of the solid, and k_B is a rate constant given by

$$k_B = \frac{4\pi}{3}\dot{G}_B^3\dot{N}_B$$

where $\dot{G}_B$ is the velocity of growth from $A \rightarrow B$ in a single dimension, and $\dot{N}_B$ is the rate of nucleation of B per unit volume. Growth of B is assumed to be spherical. To account for the fact that B can only grow into regions currently occupied by A, we rescale the unrestricted dV_A^u by the overall volume fraction of A present, to approximate the true infinitesimal change in V_A:

$$d\,V_A = \frac{V_A}{V}\,dV_A^u \tag{2}$$

Combining (1) and (2) leads to

$$\frac{dV_A}{dt} = -k_B t^3 V_A$$

It is conventional to replace t^3 by a more general factor t^{n_B-1}, where n_B is the Avrami constant, to allow for growth restricted to fewer than 3 dimensions. Thus,

$$\frac{dV_A}{dt} = -k_B t^{n_B-1} V_A \tag{3}$$

which integrates to the Avrami equation

$$V_A = V\exp[-(\mathrm{k}'_\mathrm{B}t)^{n_B}] \tag{4}$$

with $(k'_B)^{\,n_B} = k_B/n_B$.

For the process $B \rightarrow C$, it is convenient to focus on the rate of formation of C. However, the situation is still more complicated than before. One cannot simply translate the argument above to obtain

$$\frac{dV_C}{dt} = k_C t^3 V_B \tag{5}$$

because during the time period dt, the volume of species B changes due to its formation from A. In mathematical terms, V_C is a function of t and V_B, where the latter is itself

dependent on t. It follows using standard partial differentiation arguments that the total derivative is

$$\frac{dV_C}{dt} = \left(\frac{\partial V_C}{\partial V_B}\right)_t \frac{dV_B}{dt} + \left(\frac{\partial V_C}{\partial t}\right)_{V_B} \tag{6}$$

The second term on the right-hand side is the growth rate of C assuming a fixed amount of B, i.e. this is what would be obtained from the naïve argument above:

$$\left(\frac{\partial V_C}{\partial t}\right)_{V_B} = k_C t^3 V_B \tag{7}$$

The true rate of formation of C also depends on the other partial derivative,

$$\left(\frac{\partial V_C}{\partial V_B}\right)_t$$

To interpret this quantity, consider the following argument. At time t there are volumes V_A, V_B, and V_C of the three species. Had $A \rightarrow B$ been slightly faster, with $B \rightarrow C$ proceeding at the same rate as before, there would instead be volumes $V_A - dV_A$,$V_B + dV_B$, and $V_C + dV_C$. We assume that the regions containing B and C have simply increased in size at fixed composition, and hence approximate the increment dV_C by

$$dV_C = \frac{V_C}{V_B} dV_B$$

This leads to

$$\left(\frac{\partial V_C}{\partial V_B}\right)_t = \frac{V_C}{V_B} \tag{8}$$

Combining this with (6) and (7) gives

$$\frac{dV_C}{dt} = \frac{V_C}{V_B}\frac{dV_B}{dt} + k_C t^{n_C - 1} V_B \tag{9}$$

where we have again included a general Avrami constant for C formation.

Since $V_A + V_B + V_C$ is a constant, it follows that

$$\frac{dV_A}{dt} + \frac{dV_B}{dt} + \frac{dV_C}{dt} = 0$$

and so the third rate equation can be obtained from

$$\frac{dV_B}{dt} = -\frac{dV_A}{dt} - \frac{dV_C}{dt}$$

Some simple algebra with (3) and (9) leads to

$$\frac{dV_B}{dt} = \frac{k_B t^{n_B - 1} V_A - k_C t^{n_C - 1} V_B}{1 + V_C / V_B}$$

We thus model the kinetics of the reaction by the three rate equations

$$\frac{dV_A}{dt} = -k_B t^{n_B - 1} V_A \tag{10}$$

$$\frac{dV_B}{dt} = \frac{k_B t^{n_B - 1} V_A - k_C t^{n_C - 1} V_B}{1 + \frac{V_C}{V_B}} \tag{11}$$

$$\frac{dV_C}{dt} = \frac{V_C}{V_B} \frac{dV_B}{dt} + k_C t^{n_C - 1} V_B \tag{12}$$

Physically, the initial ($t = 0$) conditions are $V_A = V$, $V_B = V_C = 0$. However, the ratio V_C/V_B enters the differential equations, and it would clearly be undefined using those initial conditions. Instead, we note that the initial volumes are independent of each other, so $\left(\frac{\partial V_C}{\partial V_B}\right)_t = 0$ at $t = 0$, and hence [from (8)] we require $\frac{V_C}{V_B} = 0$ at $t = 0$. We take this as the third initial condition, in place of $V_C = 0$.

Using the initial conditions, the solution is found to be

$$\frac{V_A}{V} = \exp(-k'_B t^{n_B}) \tag{13}$$

$$\frac{V_B}{V} = \frac{1 - \exp(-k'_B t^{n_B})}{k'_C t^{n_C} + 1} \tag{14}$$

$$\frac{V_C}{V} = 1 - \frac{V_A}{V} - \frac{V_B}{V} \tag{15}$$

in which $k'_B = k_B / n_B$ and $k'_C = k_C / n_C$.

Notice that species A still obeys the Avrami equation (4): its decay is unaffected by the subsequent conversion of B to C.

Notice also that the *numerator* of (14) is what would still follow if (5) (but with a general Avrami constant) had been used instead of (9). The denominator thus corrects for the fact that B is being simultaneously created and destroyed. As time progresses, the B- and C-containing regions become more C dense, which decreases the rate of change of V_B from

what one would obtain without the correction: i.e. it tends to slow down the conversion of B to C.

Indeed, if sufficient time has passed such that $V_A \simeq 0$ then (11) becomes

$$\frac{dV_B}{dt} \simeq -\frac{k_C t^{n_C-1} V_B^2}{V}$$

This integrates to

$$\frac{V_B}{V} = \frac{n_C}{k_C t^{n_C}}$$

(using that $V_B \to 0$ as $t \to \infty$), *and hence B ultimately decays as a power of t* rather than the stretched exponential of standard Avrami kinetics, equation (4). [This conclusion also follows directly from the integrated forms of the differential equations, (9) and (10).]

S.4.2 Numerical solution of the differential equations

We fitted the experimental data in Figure 3 to the kinetic model by solving the differential equations of (10) – (12) numerically, rather than using the integrated forms of (13)—(15) directly. This was to allow for the discontinuous change in n_{COA} at $t =$ 16.5 min.

Specifically, the fits to the data for $t <$ 16.5 min, starting from initial conditions $V_A/V = 1$ and $V_C/V_B = 0$ at $t = 0$ min (as explained in the previous section), were used to obtain V_A, V_B and V_C at $t =$ 16.5 min. These values were taken as the initial conditions for the fits to the data for $t >$ 16.5 min, in which n_{COA} was changed from 1 to 2.

Both fits are shown again below (Figure S15), on both an expanded scale (top) and as a log-log plot (bottom). Note in particular that the straight-line behaviour of % COE at long times, on the log-log plot, is precisely the power-law decay highlighted at the end of the previous section. As explained there, it is a direct consequence of the fact that COE is simultaneously created and destroyed, which requires the more general mathematical treatment introduced through equation (6) [rather than the conventional equation (5)].

The model was fitted to the H_2 and D_2 data using the NonlinearModelFit function of Mathematica, assuming normally-distributed experimental measurements with common standard deviation. The function returned the best-fit parameters, along with the standard errors.

The %COD values are subject to large percentage errors when they fall below 1%, i.e. at long times, due to the precision of the experiment. The model was therefore fitted to the first

seven (ten) sets of data points for H_2 (D_2), respectively. Including all data points leads to a slightly worse fit of the model at earlier times, but does not significantly change the parameters from those above.

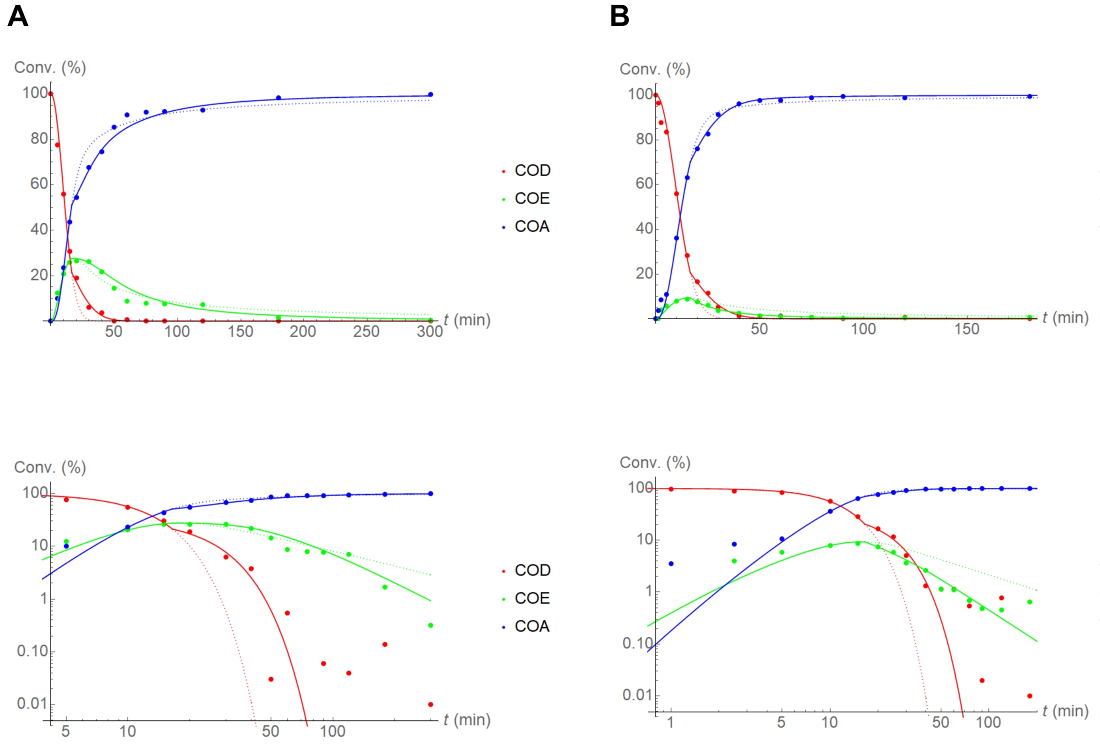

Figure S15. Sequential JMAK analysis with associated log/log plots for the solid-gas hydrogenation of **[1-COD][BAr^F$_4$]** with (A) H_2; and (B) D_2. Dotted lines show fits that <u>do not</u> include the t = 16.5 min change.

S.5 COMPUTATIONAL METHODS

Periodic DFT calculations on **[1-COA][BAr^F$_4$]** employed the CP2K program suite (Version 5.0).[8] Initial coordinates were obtained from the experimental crystallographic data, with the hydrogen positions normalised where possible with Mercury.[9] Calculations employed the Gaussian Plane Wave (GPW) formalism as implemented in the QUICKSTEP[10] module with basis sets for all atoms of double-ζ plus polarization quality in their short-range variant (DZVP-MOLOPT-SR-GTH).[11] The interaction between the core electrons and the valence shell (Rh: 17, B: 3, C: 4, P: 5, F: 7, H: 1 electrons) was described by Goedecker-Teter-Hutter (GTH) pseudo potentials.[12-14] The generalized gradient approximation (GGA) to the exchange-correlation functional according to Perdew-Burke-Ernzerhof (PBE)[15] was used in combination with Grimme's D3-correction for dispersion interactions.[16] The auxiliary plane wave basis set was truncated at a cutoff of 500 Ry. The maximum force convergence criterion was set to 10^{-4}

Eh·Bohr^{-1}, whilst default values were used for the remaining criteria. The convergence criterion for the self-consistent field (SCF) accuracy was set to 10^{-7} Eh and 10^{-8} Eh for geometry optimizations and vibrational analysis, respectively.

The Brillouin zone was sampled using the Γ-point. Periodic boundary conditions (PBC) were applied throughout in combination with fixed unit cell parameters obtained from experiment. All geometries were first partially relaxed, keeping the heavy atoms (non-H, F) fixed, then fully relaxed without imposing any constraints, whilst keeping unit cell parameters constant in all cases. The fully optimised geometry was further characterized by analysis of the numerical second derivatives with a displacement of 0.01 Bohr, and found to have no imaginary eigenvalues. Cartesian coordinates of computed structure are included below.

The topology of the electron density of the **[1-COA]$^+$** cation was analysed by means of QTAIM (Quantum Theory of Atoms in Molecules),[17] as implemented in the AIMALL package.[18] Inner shell electrons on Rh and P modelled by ECPs were represented by core density functions (extended wavefunction format). NBO calculations were performed using the NBO 6.0 program,[19] using the same geometries as for the QTAIM calculations above. NCI calculations were performed using the NCIPLOT program[20,21]. The promolecular electron density was employed. Geometries of the **[1-COA]$^+$** cation and the nearest-neighbour ion-pair were extracted from the CP2K optimised geometries. Orbital plots were created with Chemcraft[22] with an outer contour value of 0.07465.

S.5.1 QTAIM study of [1-COA]$^+$

(a)

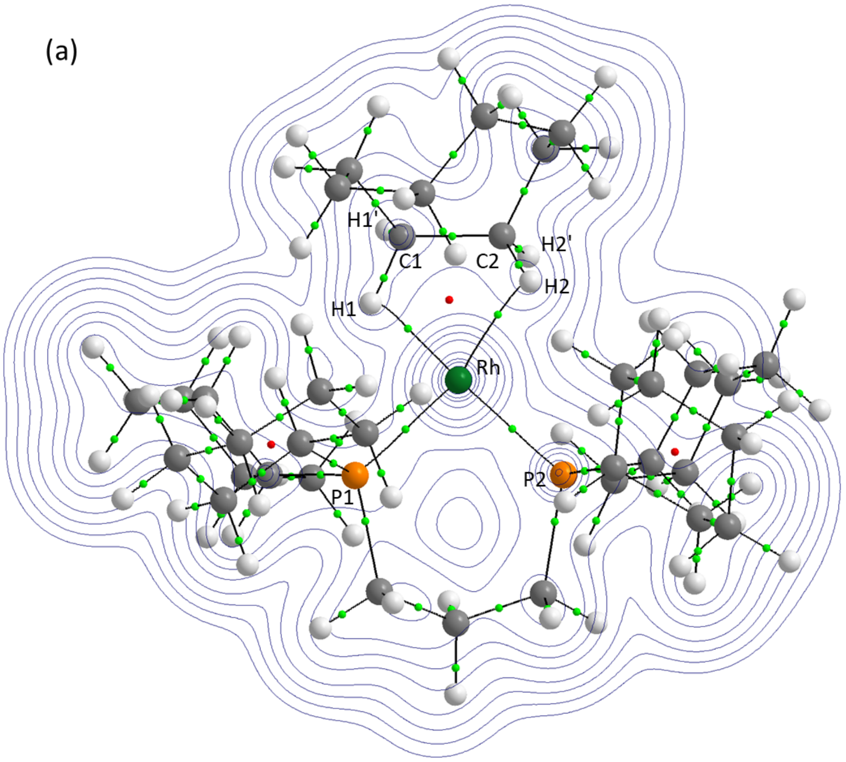

(b)

Bond	Distance/Å	r(r)	∇^2r(r)	e	H(r)
**Rh–H1**	1.890	0.061	+0.232	0.971	-0.077
**Rh–H2**	1.896	0.056	+0.218	0.667	-0.071
**C1–H1**	1.157	0.233	-0.613	0.043	-0.251
**C1–H1'**	1.096	0.278	-0.953	0.010	-0.327
**C2–H2**	1.154	0.232	-0.606	0.009	-0.252
**C2–H2'**	1.100	0.275	-0.933	0.005	-0.321
**Rh–P1**	2.219	0.116	+0.080	0.029	-0.130
**Rh–P2**	2.223	0.115	+0.082	0.022	-0.128
**C1–C2**	1.544	0.115	-0.496	0.008	-0.239

Figure S16. (a) Molecular graph for the **[1-COA]$^+$** cation with bond critical bonds (BCPs) in green and ring critical points (RCPs) in red. Electron density contours are shown in the plane containing Rh, H1 and H2 (b) Selected metrics for key BCPs (atomic units unless otherwise stated).

3.5.2 Non-covalent interaction (NCI) study of the [1-COA][$BArF_4$] ion-pair

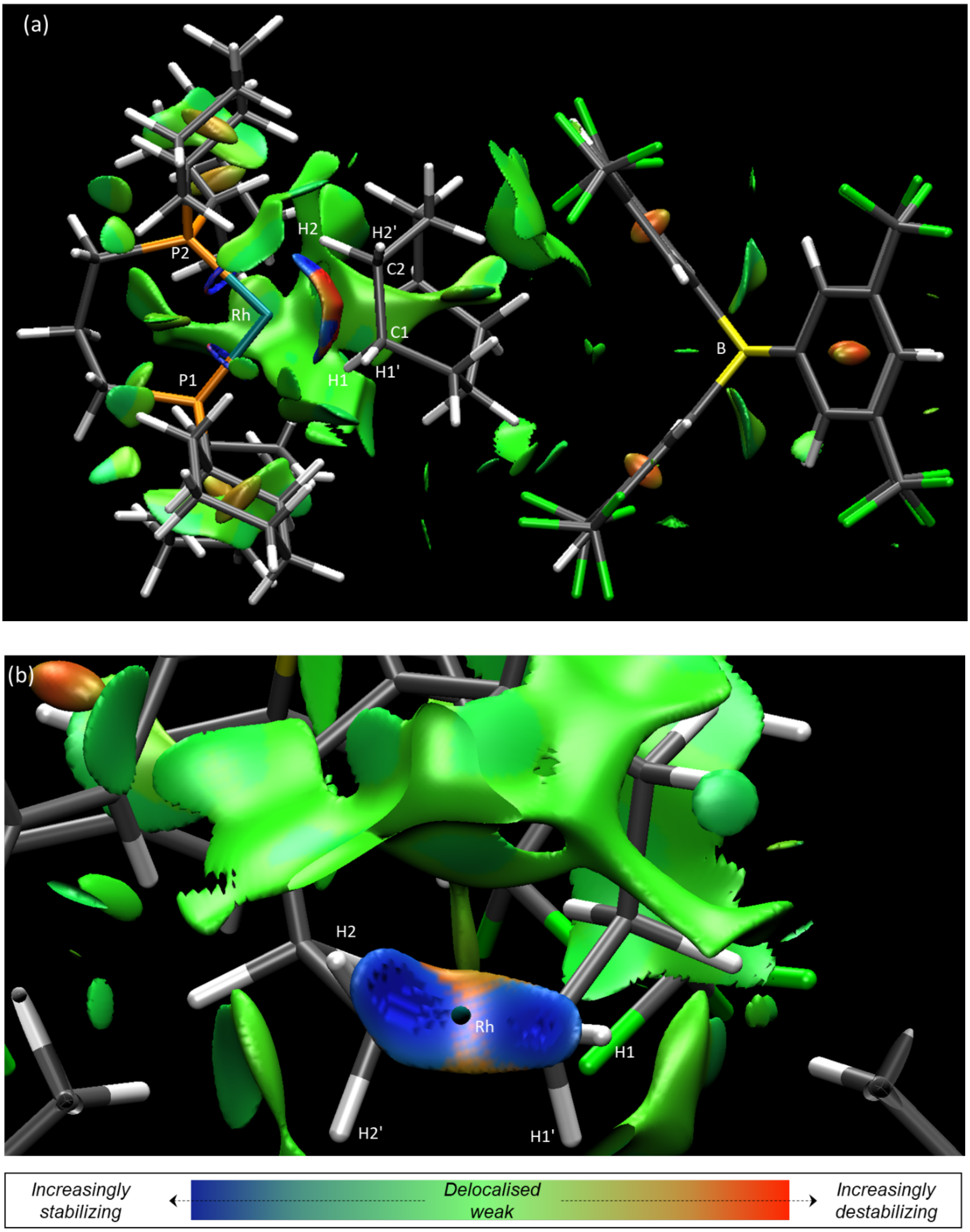

Figure S17. Two views of the NCI plots for the [1-COA][BAr^F_4] ion-pair (a) View from above the COA ligand showing both the cation and anion (b) Detail viewed from the Rh center looking down an axis passing through the center of the C1-C2 bond. Isosurfaces are generated for σ = 0.3 au and −0.07 < ρ < 0.07 au; a key showing the color scheme employed is also provided.

S.5.3 Natural bond orbital analysis of the $[1\text{-COA}]^+$ cation

(a)

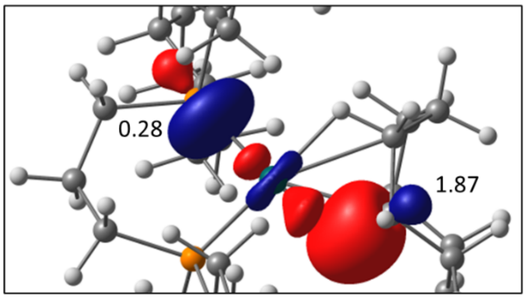

$\sigma_{C1\text{-}H1} \rightarrow \sigma^*_{Rh\text{-}P2}$ = 17.8 kcal/mol

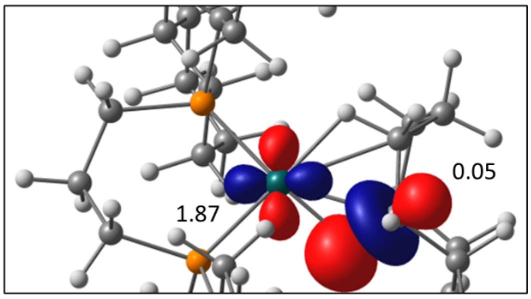

$Rh_{LP} \rightarrow \sigma^*_{C1\text{-}H1}$ = 3.2 kcal/mol

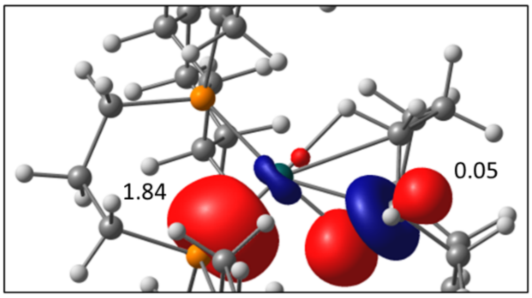

$\sigma_{Rh\text{-}P1} \rightarrow \sigma^*_{C1\text{-}H1}$ = 3.0 kcal/mol

Other contributions:
$Rh_{LP} \rightarrow \sigma^*_{C1\text{-}H1}$ = 1.4 kcal/mol
$Rh_{LP} \rightarrow \sigma^*_{C1\text{-}H1}$ = 1.1 kcal/mol
$\sigma_{Rh\text{-}P1} \rightarrow \sigma^*_{C1\text{-}H1}$ = 1.3 kcal/mol

Total back-donation = 10.0 kcal/mol

(b)

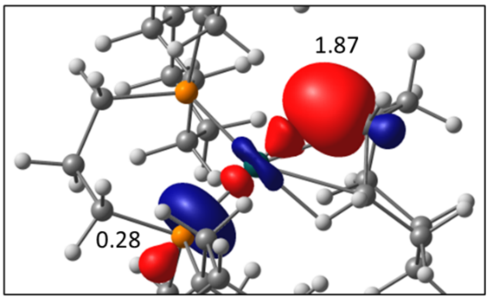

$\sigma_{C2\text{-}H2} \rightarrow \sigma^*_{Rh\text{-}P1}$ = 17.5 kcal/mol

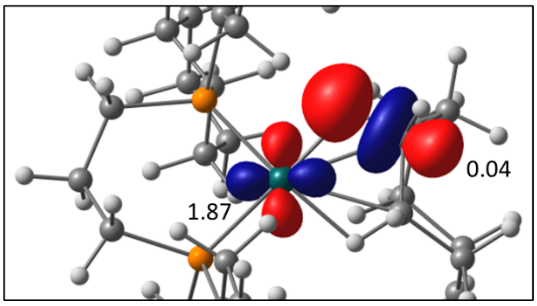

$Rh_{LP} \rightarrow \sigma^*_{C2\text{-}H2}$ = 2.9 kcal/mol

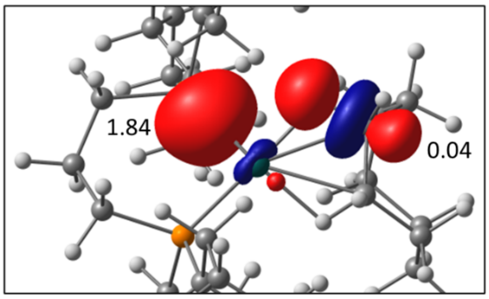

$\sigma_{Rh\text{-}P2} \rightarrow \sigma^*_{C2\text{-}H2}$ = 3.8 kcal/mol

Other contributions:
$Rh_{LP} \rightarrow \sigma^*_{C1\text{-}H1}$ = 0.1 kcal/mol
$Rh_{LP} \rightarrow \sigma^*_{C1\text{-}H1}$ = 0.4 kcal/mol
$\sigma_{Rh\text{-}P1} \rightarrow \sigma^*_{C1\text{-}H1}$ = 1.3 kcal/mol

Total back-donation = 8.5 kcal/mol

Figure S18. Major donor−acceptor interactions derived from the second-order perturbation NBO analysis (kcal/mol); (a) involving C1-H1; (b) involving C2-H2. NBO occupations are also indicated and well as other minor contributions to the back donations.

S.5.4 Computed energy for [1-COA][BAr^F_4]

E(SCF) = -2272.32830840 Hartrees

S.6 References

1 Martínez-Martínez, A. J.; Tegner, B. E.; McKay, A. I.; Bukvic, A. J.; Rees, N. H.; Tizzard, G. J.; Coles, S. J.; Warren, M. R.; Macgregor, S. A.; Weller, A. S., *J. Am. Chem. Soc.* **2018,** *140*, 14958-14970.

2 Fulmer, G. R.; Miller, A. J. M.; Sherden, N. H.; Gottlieb, H. E.; Nudelman, A.; Stoltz, B. M.; Bercaw, J. E.; Goldberg, K. I. *Organometallics* **2010**, *29*, 2176-2179.

3 Hu, B.; Gay, I. D. *Langmuir* **1999**, *15*, 477-481.

4 Palatinus, L.; Chapuis, G. *J. Appl. Crystallogr.* **2007**, *40*, 786-790.

5 Sheldrick, G. M., *Acta Crystallogr. Sect. A* **2008**, *64*, 112-122.

6 Dolomanov, O. V.; Bourhis, L. J.; Gildea, R. J.; Howard, J. A. K.; Puschmann, H., *J. Appl. Crystallogr.* **2009**, *42*, 339-341.

7 Spek, A.L. *Acta Crystallogr. Sect. C* **2015**, *71*, 9-18.

8 Hutter, J.; Iannuzzi, M.; Schiffmann, F.; VandeVondele, J. *Wires Comput. Mol. Sci.* **2014**, *4*, 15.

9 Mercury CSD 2.0, Macrae, C. F.; Bruno, I. J.; Chisholm, J. A.; Edgington, P. R.; McCabe, P.; Pidcock, E.; Rodriguez-Monge, L.; Taylor, R.; van de Streek, J.; Wood, P. A. *J. Appl. Cryst.*, **2008**, *41*, 466-470.

10 Van de Vondele, J.; Krack, M.; Mohamed, F.; Parrinello, M.; Chassaing, T.; Hutter, J. *Comput. Phys. Commun.* **2005**, 167, 103–128.

11 VandeVondele, J.; Hutter, J. *J. Chem. Phys.* **2007**, *127*, 114105.

12 Hartwigsen, C.; Goedecker, S.; Hutter, J. *Phys. Rev. B* **1998**, *58*, 3641.

13 Goedecker, S.; Teter, M.; Hutter, J. *Phys. Rev. B* **1996**, *54*, 1703.

14 Krack, M. *Theor. Chem. Acc.* **2005**, *114*, 145.

15 Perdew, J. P.; Burke, K.; Ernzerhof, M. *Phys. Rev. Lett.* **1996**, *77*, 3865.

16 Grimme, S.; Antony, J.; Ehrlich, S.; Krieg, H. *J. Chem. Phys.* **2010**, *132*, 154104.

17 Bader, R. F. W. *Atoms in Molecules: A Quantum Theory*; Oxford University Press, 1990.

18 AIMAll (Version 17.11.14), Todd A. Keith, TK Gristmill Software, Overland Park KS, USA, 2017 (aim.tkgristmill.com)

19 NBO 6.0. E. D. Glendening, J. K. Badenhoop, A. E. Reed, J. E. Carpenter, J. A. Bohmann, C. M. Morales, C. R. Landis, and F. Weinhold (Theoretical Chemistry Institute, University of Wisconsin, Madison, WI, 2013); http://nbo6.chem.wisc.edu/

20 E. R. Johnson, S. Keinan, P. Mori-Sanchez, J. Contreras-Garcia, A. J. Cohen, and W. Yang, *J. Am. Chem. Soc.* **2010**, *132*, 6498-6506.

21 J. Contreras-Garcia, E. R. Johnson, S. Keinan, R. Chaudret, J-P. Piquemal, D. N. Beratan, and W. Yang. *J. Chem. Theory Comput.* **2011**, *7*, 625-632.

22 Chemcraft - graphical software for visualization of quantum chemistry computations. https://www.chemcraftprog.com
